# Language about the future on social media as a novel marker of anxiety and depression: A big-data and experimental analysis

**DOI:** 10.1016/j.crbeha.2023.100104

**Published:** 2023

**Authors:** Cole Robertson, James Carney, Shane Trudell

**Affiliations:** a Emory University; b The London Interdisciplinary School; c Nightingale Counselling and Research

**Keywords:** Future time reference, Temporal discounting, Mood disorders, Anxiety, Depression, Big-data, Time perspective

## Abstract

Anxiety and depression negatively impact many. Studies suggest depression is associated with future time horizons, or how “far” into the future people tend to think, and anxiety is associated with temporal discounting, or how much people devalue future rewards. Separate studies from linguistics and economics have shown that how people refer to future time predicts temporal discounting. Yet no one—that we know of—has investigated whether future time reference habits are a marker of anxiety and/or depression. We introduce the FTR classifier, a novel classification system researchers can use to analyse linguistic temporal reference. In Study 1, we used the FTR classifier to analyse data from the social-media website Reddit. Users who had previously posted popular contributions to forums about anxiety and depression referenced the future and past more often than controls, had more proximal future and past time horizons, and significantly differed in their linguistic future time reference patterns: They used fewer future tense constructions (e.g. *will*), fewer high-certainty constructions (*certainly*), more low-certainty constructions (*could*), more bouletic modal constructions (*hope*), and more deontic modal constructions (*must*). This motivated Study 2, a survey-based mediation analysis. Self-reported anxious participants represented future events as more temporally distal and therefore temporally discounted to a greater degree. The same was not true of depression. We conclude that methods which combine big-data with experimental paradigms can help identify novel markers of mental illness, which can aid in the development of new therapies and diagnostic criteria.

## Highlights

• Language on social media by suffers of anxiety and/or depression differed from healthy controls.
• Anxious/depressed posts referred to non-present time more and used more proximal time frames.
• Anxious/depressed posts used more low-certainty, and more bouletic (about desires) language.
• Anxious–but not depressed–participants construed future events as more temporally distal.
• This caused anxious–but not depressed–participants to devalue delayed outcomes relatively more.

## 1 Introduction

Millions of people suffer from mental illness world wide. For example, in North America the prevalence of mental health disorders in adults may be as high as 20% (National Institute of Mental Health, 2019). In addition to the impacts on well-being—important in their own right—the estimated economic costs of mental illness in North America are as much as US$83.1bn annually (Dewa and McDaid, 2011). At the same time, medical science has struggled to predict the onset of mental illness from physiological markers which has made preventative medicine difficult to implement (Kapur et al., 2012). A potentially promising avenue is to identify linguistic markers of mental illness. One way to do this is using naturalistic linguistic data from social-media websites. This has lead to work which uses social-media data to predict various aspects of personality, including the onset and diagnosis of mental illness (see Goldstone, Lupyan, 2016, Guntuku, Yaden, Kern, Ungar, Eichstaedt, 2017).

For instance, the language used in previous posts on the social-media site Reddit was successfully used to predict whether a user would later post to a forum dedicated to mental illness (Thorstad and Wolff, 2019a). Tweets by Twitter users who self-diagnosed as suffering from depression or post traumatic stress disorder were successfully classified above chance levels (Coppersmith et al., 2015). Additionally, posts to 11 different Reddit forums dedicated to mental illness were successfully differentiated with an average of 72% precision (Gkotsis et al., 2017).

Many predictive studies have implemented “black-box” machine learning algorithms which focus on predicting mental health outcomes from prior social-media data. While predictive approaches are undoubtedly valuable, model parameters are not always interpretable by human practitioners (Rai, 2020). Additionally, black-box approaches risk amplifying markers of more general structural inequality that can be associated with mental illness (see Eubanks, 2018, Obermeyer, Powers, Vogeli, Mullainathan, 2019). Another strand of research therefore seeks to complement predictive approaches with descriptive ones which attempt to generate psychological insights from the analysis of social-media data. For instance, Thorstad and Wolff (2019a) used per-word model weights to understand which words were most predictive of a user later posting to a Reddit forum focused on depression. Similarly, De Choudhury et al. (2014) found language use differences on Facebook posts as a function of whether survey respondents had been clinically diagnosed with postpartum depression.

In the present study we therefore implemented a hybrid methodology, using a combination of machine learning and descriptive natural language processing to explore the relationships between future time horizons, linguistic Future Time Reference (FTR) patterns, and anxiety and depression. To do this, we developed a novel classification system. It has two stages, first estimating whether an item of text data refers to the future or past (“time-reference” classification). Then, for future-referring data, it estimates *how* the future is characterised using a simple set of semantic categories based on the linguistic literature on future time reference (“FTR-type” classification). The choice of these variables was motivated by previous research which suggests that how people construe future events—their future time perspective—is a marker of anxious and/or depressive tendencies (Dilling, Rabin, 1967, Pulcu, Trotter, Thomas, McFarquhar, Juhasz, Sahakian, Deakin, Zahn, Anderson, Elliott, 2014, Steinglass, Lempert, Choo, Kimeldorf, Wall, Walsh, Fyer, Schneier, Simpson, 2017).

Future time perspective encompasses how people think about and relate to future events. It includes questions such as: Do people tend to save for the future or spend resources now, do they conceive of future events as proximal or distal, and do they tend to imagine temporally proximal or distal future events? Measures of future time perspective have been found to be significant markers of anxiety (Papastamatelou et al., 2015), major depressive disorder (Pulcu et al., 2014) and social anxiety disorder (Steinglass et al., 2017).

Particularly, people suffering from depression had contracted “future time horizons” relative to controls (Dilling and Rabin, 1967). This is usually measured using the Wallace task (Wallace, 1956). Participants are asked to think of 10 events that are likely to occur in their future lives, and what age they expect to be when the events will occur. Their future time horizon is then calculated by subtracting their current age from the ages they give. This approximates how far into the future participants tend to imagine themselves.

Pathological gamblers (Hodgins and Engel, 2002), as well as people suffering from alcoholism (Smart, 1968) and substance abuse (Petry et al., 1998) problems were all found to have shortened future time horizons compared with healthy controls. While these studies do not explicitly study depression, substance abuse disorders and depression co-occur often enough to warrant potential connections between these results and underlying depressive tendencies (Barrault, Varescon, 2013, Blaszczynski, McConaghy, 1989, Felton, Strutz, McCauley, Poland, Barnhart, Lejuez, 2020). Since anxiety tends to involve excessive “anticipation of future threat” (American Psychiatric Association, 2015, p. 1), the subjective experience of future events seems almost inherently salient to the experience of anxiety. However, the relationship between anxiety and future time perspective seems less well understood then for depression.

Most research into anxiety and time perspective has used temporal discounting paradigms. Temporal discounting captures the extent to which people devalue delayed future outcomes as a function of the length of time until they will occur. In general, people tend to devalue delayed rewards as a function of wait time, but the extent of such devaluation differs between individuals (Green and Myerson, 2004). These differences are usually measured by giving participants a series of binary choices between immediate and delayed rewards, for instance $10 now vs. $20 in a month. “Present-oriented” people tend to devalue future outcomes and would therefore opt for the immediate $10, while “future-oriented” people tend to devalue less steeply and would therefore opt to wait for the $20. The term “time preferences” refers to individual differences in tasks like this. These measures are not unrelated to time horizons. Shorter time horizons tend to be associated with increased discounting (Thorstad, Nie, Wolff, 2015, Thorstad, Wolff, 2018), and researchers increasingly understand temporal discounting processes in terms of subjective representations of future time, as opposed to discounting rates over objective time (Kim, Zauberman, 2009, Zauberman, Kim, Malkoc, Bettman, 2009).

While people suffering from major depressive disorder have been found to temporally discount more than healthy individuals (Felton, Strutz, McCauley, Poland, Barnhart, Lejuez, 2020, Pulcu, Trotter, Thomas, McFarquhar, Juhasz, Sahakian, Deakin, Zahn, Anderson, Elliott, 2014), the results around anxiety have been mixed. Some findings suggest people suffering from social anxiety discount more (Rounds et al., 2007), while others report the effect goes in the opposite direction (Steinglass et al., 2017), or that there is no significant relationship (Jenks and Lawyer, 2015). This suggests more research may be needed to untangle how future time perspective may relate to the experience of anxiety.

An understudied topic that may help to resolve these issues is the linguistics of how people talk about the future. A growing body of research indicates that linguistic FTR may be an important factor in shaping future time perspective. FTR is a catch-all term used for any linguistic statement which refers to future events, whether or not it uses the future tense (Dahl, 1985, Dahl, 2000). For instance, *It could rain tomorrow, It will rain tomorrow*, and *I hope it rains tomorrow* all involve FTR. Recent work suggests that cross-linguistic differences in FTR grammar impact speakers’ time preferences. Chen (2013) provides a framework for how this might occur, hypothesising that speaking languages which oblige speakers to use the future tense for FTR causes speakers to perceive the future as farther away and therefore to discount more. For example, in English, speakers are obliged to use *will* in the sentence *It will rain tomorrow*. On the other hand, speakers of Dutch are free to use the present tense, *Het regent morgen*, ‘It rains tomorrow.’ Chen (2013) theorised that consistently using the present tense for FTR would collapse future into present time, effectively causing speakers to construe objective future dates as subjectively more proximal and therefore temporally discount less (i.e. be more willing to wait for subjectively more proximal future rewards). In support of this hypothesis, he found that speakers of languages like Dutch were more likely to have saved each year, and retired with more assets. Since then, numerous other studies have found that speakers of languages like Dutch tend to behave as though they discount less (e.g. Chi, Su, Tang, Xu, 2018, Falk, Becker, Dohmen, Enke, Huffman, Sunde, 2018, Figlio, Giuliano, Özek, Sapienza, 2016, Liang, Marquis, Renneboog, Sun, 2018, Mavisakalyan, Tarverdi, Weber, 2018, Pérez, Tavits, 2017, Zhu, Hu, Wang, Zheng, 2020).

However, in addition to constraints on future tense use, cross-linguistic differences in FTR grammar can involve constraints on the encoding “modal” notions of possibility, probability, and certainty. For instance, it is not actually obligatory to use *will* in the sentence *It will rain tomorrow*. Rather, English grammar obliges speakers to use one of *It could/may/might/should/shall/would/is going to rain tomorrow*. These “modal” verbs generally encode notions of weakened certainty relative to the future tense constructions (*shall, will,* and *be going to*). In previous research, we found obligatory use of low-certainty modals for FTR in English drove differences in time preferences involving risky outcomes between English and Dutch speakers. This suggests that FTR usage habits reflect latent beliefs about the risk and/or temporal distance of future events, and are related to time perspective.

However, the relationship between FTR habits and anxiety and depression has not been studied, not least because of the difficulty involved in identifying and characterising loosely grammatically marked FTR statements in English. For instance, *The train gets in at 7 tonight*, is present tense but makes FTR; *That will be the postman* (said on hearing a knock at the door) utilises the nominal future tense *will* but makes present time reference; *It could rain tomorrow* is present tense but makes FTR (Condoravdi, 2002), and *He could run a 4 min mile in his 20s* uses the same future-shifting modal verb but makes past time reference. We therefore developed an FTR classification system which helps researchers solve these problems. We used it to conduct an exploratory study which used big-data methodology to understand whether people who had posted to Reddit forums dedicated to anxiety and depression differed from a random sample in terms of their FTR language use and future time horizons. There is little precedent in the literature for how this set of variables may affect one another, beyond the basic observation that time perspective seems related to anxiety and depression. As such, we made that limited hypothesis that FTR habits and temporal horizons would be different in anxious and/or depressed individuals as compared to a randomly selected control sample.

## 2 Study 1

The basis of Study 1 is an analysis of data downloaded from Reddit. Reddit is a social media platform with over 430m average monthly users (Reddit Inc., 2020). The site is similar to a message board or traditional forum. Users (“redditors”) communicate in forums (“subreddits”) dedicated to specific topics. There are no character limits to user contributions (“posts”), so users are free to comment in as much detail as they wish. Subreddits including topic focus and rules on acceptable behaviour are created and self-monitored by moderator teams comprising leading subreddit members. Comments are “upvoted” and “downvoted”, and can be sorted by various criteria, for instance most upvoted of all time. The names of subreddits usually reflect their topical focus and are prefaced with *r/*, for instance, *r/news* is dedicated to sharing US and international news stories.

The largest subreddits dedicated to anxiety and depression are *r/Anxiety* and *r/depression*. In December, 2020, *r/Anxiety* had 417k members, and *r/depression* had 712k members (Reddit Inc., 2020). Both are focused on providing support and advice for people dealing with issues related to anxiety and depression (respectively) and both allow posts by sufferers or those close to them.

### 2.1 Methods and materials

This study was approved by the internal review board of Brunel University London (Ref. 7863-A-Jan/2018-10690-1).

#### 2.1.1 Data acquisition

We were concerned that language used when posting to *r/Anxiety* and *r/depression* might differ from a random sample not due to the habits of speech of the posters, but due to rules of discourse imposed by moderators. We therefore implemented a “snowball” sampling approach, first constructing a “seed” sample of redditors who had posted to *r/Anxiety* and *r/depression*, and then a final sample comprising these same redditors’ top posts in any subreddit.

Specifically, we used the Python Reddit API Wrapper (Boe et al., 2020), to download the 1,000 “hottest” comments in each of *r/Anxiety* and *r/depression*. Here “hotness” is defined by a Reddit proprietary algorithm for ranking content. It is calculated by taking the $log_{10}$ of the net number of upvotes and adding it to the number of 12.5 h periods that have passed since the first ever Reddit post. This means that, for each successive 12.5 h period, a post must get ten times the number of upvotes than it had in the previous 12.5 h period to retain the “hot” score it had when it was 12.5 h younger. Its value is that it captures topical content specific to a given subreddit, where other measures like “top”, which counts total number of upvotes, are disproportionately captured by “stickied” content that is permanently visible.

This resulted in a sample of n=910 posts from *r/Anxiety* and n=902 posts from *r/depression*. (As scores for user-deleted submissions are still returned by the API, the number of usable posts was less than 1,000.) We then downloaded up to the top 1,000 most recent posts made to any subreddit by each unique redditor in these “seed” data. These data were downloaded 30 October 2019. This resulted in n=224,009 posts made by redditors who had once posted popular content in *r/Anxiety* (the “anxiety” condition), and n=182,555 posts made by redditors who had once posted popular content in *r/depression* (the “depression” condition). However, we found no differences between the anxiety and depression condition on any measure in this study, and so combined these into a single “mental health” condition. We constructed a control sample of n=213,132 posts by repeating an identical process with the *r/All* subreddit, which is a compendium of all content generated on Reddit. These data were downloaded on 1 February 2020. We refer to the contrast between the mental health and control conditions as “data-source condition”.

This meant our final sample comprised N=619,696 posts, drawn from N=13,651 unique subreddits (n=6,978 [mental health], n=2,781 [control], and n=3,856 crossover subreddits which had been posted to in both the mental health and control conditions), and N=4,205 unique redditors (n=1,810 [mental health], n=2,395 [control], no crossovers). It should be noted that this process resulted in some posts from the original seed subreddits still being retained in the final sample (n=10,145 posts from *r/Anxiety*, and n=17,319 from *r/depression*). Excluding these data did not substantively change any results, i.e. cause significance to cross critical thresholds or coefficients to change sign, so we did not exclude them.

#### 2.1.2 Data preprocessing

Inspection of the data indicated that, due to their unrestricted length, a number of posts contained references to both past and future time. We therefore split posts into constituent sentences using a non-monotonic transition system dependency parsing algorithm (Honnibal and Johnson, 2015). This is essentially a probabilistic sentence boundary detector implemented in Python (Python Software Foundation, 2017) in the open-source natural language processing package *spaCy* (Explosion AI, 2020). This resulted in N=2,014,181 sentences (n=1,549,023 [mental health], n=465,158 [control]).

#### 2.1.3 Temporal horizons

To estimate temporal horizons, we used the Stanford University Time (SUTime) temporal tagging system (Chang and Manning, 2012). SUTime is a rule-based deterministic natural language tagging system which uses regular expressions which combine both keywords (e.g. “yesterday”) and rules (e.g. “[DATE] at [TIME]”) to identify temporal expressions and convert them to numerical data. It processes absolute dates (e.g. November 7, 2012), relative dates (e.g. “this Friday”), and combinations of these (e.g. “tomorrow at 3pm”). For relative dates, we used time of posting (available from the Reddit API) as the reference date. For instance, if a redditor referenced “tomorrow” on 10-06-2020, SUTime would return 11-06-2020. To calculate time horizons (days), we subtracted time of posting from the SUTime reference values. In cases where there was more than one time reference in one sentence, we took the mean, i.e. $H\left(s\right)=\sum_{r}\frac{D(r)}{n}$; where H(s) is the time horizon for a given s sentence, r is a temporal reference, D(r) is the number of days from time of posting, and n is the number of temporal references in the sentence (Thorstad and Wolff, 2018). Such methods with data from Twitter have been found to positively and significantly correlate with participants’ future time horizons as measured by the Wallace task, as well as predict both collective and individual intertemporal decision making (i.e. temporal discounting) (Thorstad and Wolff, 2018).

#### 2.1.4 The FTR classifier

**Fig. 1:**
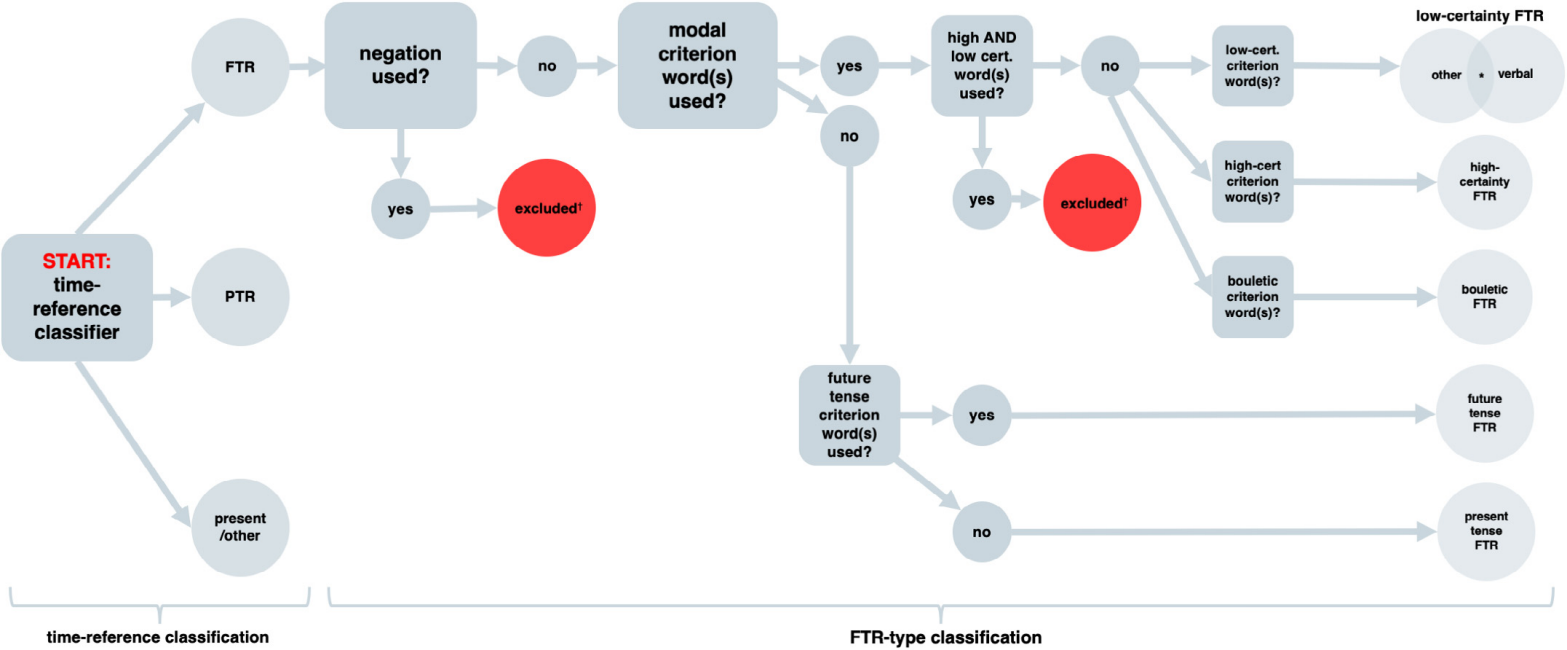
A schematic flow diagram of the FTR classification system, including time reference classification (the Prodigy model), and the closed-vocabulary FTR-type classifier. Modal categories “dominate” tense categories, so that examples such as *It willpossibly rain tomorrow* or *Rain tomorrow isprobable*, are classed as other-low-certainty and not also future/present tense. ^⁎^Overlap between verbal and other modal category Venn diagrams: In cases of so-called “modal concord”, it is possible to use both a modal periphrastic modifier and a modal verb, e.g. *It could probably rain tomorrow* (Huitink, 2012). Such responses are classified as *both* other- and verbal-low-certainty. ^†^ Exclusions: Both negations and what we term “mixed modal” responses make it impossible for keyword techniques to identify the intended modal polarity. For instance, in the presence of negations, modal adverbs reverse modal polarity, e.g. *I am not certain it will rain tomorrow*, would be classed as other-high-certainty because of the use of *certain*, but it expresses low certainty. What we term “mixed modal” responses are similarly complex. These are expressions which use both high- and low-certainty keywords, e.g. *There is definitely a possibility of rain tomorrow.* The FTR-type classifier cannot “know” which word should be used as the class criterion in such cases. However, there were no such cases in our data.

To estimate both *whether* and *how* comments referred to the past or future, we built a classification system we refer to as the FTR classifier. It involved two stages: first, a machine-learning model estimates whether an item of text data refers to the past, future, or present/other^1^; second, FTR responses are passed to the FTR-type classifier which characterises *how* the future is referenced, see Fig. 1. The FTR classifier is freely available to be used and can be installed as a Python package, (see Supplementary Materials [SM] available at https://osf.io/nqh9r/).

*Time reference classification* To estimate whether sentences were FTR or Past Time Reference (PTR), we used an active learning paradigm implemented in Prodigy (Montani and Honnibal, 2020) to train a machine learning “time reference” classifier. Active learning is a way of reducing the number of annotated examples machine learning models need to generate reliable predictions in supervised learning paradigms. In supervised learning, human raters annotate data and machine learning models are then trained to predict annotations from input data (in this case raw text). Active learning speeds up this process by keeping a machine learning model “in the loop” during the annotation process, and selecting the un-annotated data about which the model is most uncertain. This increases the number of annotations which are near the boundary criteria of the unknown function the model is attempting to approximate, and reduces the number of annotations the model is already able to confidently predict, thereby reducing the total number annotations needed.

Prodigy was used to annotate sentences for both FTR (n=4002 [$n_{accept}=1403$, $n_{reject}=2406$, $n_{ignore}=193$^2^]), and PTR (n=2382 [$n_{accept}=660$, $n_{reject}=1704$, $n_{ignore}=18$]). Because the active learning process resulted in overlaps, there were N=4,947 unique text examples. For PTR, all annotations were drawn from our Reddit data. FTR annotations were drawn from a combination of a dataset of Tweets and Reddit data (see SM). Because we wanted to cast as wide a net as possible to better understand linguistic usage within the broad notional domains of future and past time reference, our annotation criteria were semantic rather then formal. Any statement which made FTR/PTR was classed positively, regardless of formal tensing. For instance, *They could win, It will be ok, It rains tomorrow, I go in three weeks, My train is arriving soon*, and (in November) *Christmas is soon* would all be classed as FTR. Similarly, *It has rained, I saw her, It was tiresome, I’m walking along the other day*, and *I’m tired from my run this morning* would all be classed as PTR.

Using Prodigy, we trained a model on these data using 80%/20% train/evaluate split, 10 iterations over the data, a dropout rate of 0.2, and exclusive categories.^3^ The Prodigy text classification system wraps a *spaCy* ensemble model comprising a bag of words model and a 1-dimensional Convolution Neutral Net (CNN) where token vectors are calculated using a CNN, mean pooled and used as features in a feed-forward network (Explosion AI, 2020). This is *spaCy*’s out-of-the-box text classification system, and essentially combines a simple bag-of-words model which classifies on the basis of unordered words, with a more complex neutral network (the CNN). Because not all the FTR annotations had been drawn from the Reddit data and we wanted to make sure the model’s out of sample performance was acceptable, we annotated two additional “test” datasets drawn exclusively from the Reddit data ($N_{FTR}=477$, and $N_{PTR}=489$). The model outputs scalar probabilities that a sentence is FTR or PTR. We converted these to categorical data by splitting on p=0.5:

(1) $$f\left(p_{tr}\right)=\left\{ \begin{array}{cc} 1, & \text{if}\;p_{{}_{TR}}\geq 0.5\\ 0, & \text{otherwise} \end{array}\right.$$

where $p_{{}_{TR}}$ is the probability p that an item of text data is either FTR or PTR. This allowed us to test accuracy against categorical human annotations. As shown in Table 1, model performance on the withheld data was relatively high for both FTR and PTR, though slightly higher for FTR than PTR, for reasons that are not entirely clear. Low recall for PTR classifications indicates that the model tends to miss some true PTR statements. This is because we prioritised precision; High precision indicates that when the model does predict PTR it is correct 83% of the time. We reasoned that having reliable positive predictions was more important than identifying a high percentage of true positives.

**Table 1:** Time reference and FTR-type classifier performance metrics in Study 1.

classifier	category	sub-cat.	accuracy	precision	recall	*F1*
time reference	FTR	-	0.91	0.93	0.73	0.82
	PTR	-	0.81	0.83	0.35	0.49
FTR type	future tense	-	0.84	0.81	0.92	0.86
	present tense	-	0.93	0.94	0.99	0.96
	low-certainty	verbal	0.99	>0.99	0.99	0.99
		other	0.96	>0.99	0.97	0.98
	deontic modality	-	0.98	>0.99	0.98	0.99
	high-certainty	-	0.98	0.99	0.98	0.99
	bouletic modality	-	0.94	0.99	0.94	0.97
	irrealis	-	0.89	0.99	0.89	0.94

Accuracy is defined as a=(tp+tn)/(tp+fp+fn+tn) where tp is the number of true positives, tn is the number of true negatives, fp is the number of false positives and fn is the number of false negatives. Accuracy captures the classifier’s performance without prioritising either positive or negative examples. Precision is p=tp/(tp+fp); it captures the model’s likelihood of being correct if it makes a positive prediction and is therefore sensitive to the model’s type I error rate. Conversely, denominator in recall is the false negatives, r=tp/(tp+fn); it therefore captures whether the model tends to miss true examples, and is sensitive to the model’s type II error rate. *F1* is the harmonic mean of recall and precision, *F1*=(2rp)/(r+p), and attempts to balance the two.

#### 2.1.5 FTR-type classification

With the sentences referring to the future now identified, a training set was now in place for building a classifier which differentiated the various ways in which it is possible to refer to the future in English. To establish this, we developed the FTR-type classifier. It is closed-vocabulary deterministic keyword-based classification program written in Python (Python Software Foundation, 2017). It classifies short future-referring text documents into 7 exclusive categories based on the presence of keywords which encode semantic domains most salient to FTR. One of these categories is further broken down according to the word classes of the keywords involved. Each category is dichotomous, and coded (1) for positive examples, otherwise coded (0).

Two categories are tense-based and the other 5 are based on modal semantics. Modality generally involves quantifying what is possible and/or necessary relative to any of several common modal “bases” (Palmer, 2001). For instance, epistemic modality involves speakers expressing what they think is probable or unlikely relative to an epistemic “base”, i.e. what they know and believe about the world (Nuyts, 2000); bouletic modality involves speakers quantifying what they think is possible or desirable relative to a desiderative base, i.e. what they hope or want to occur (Palmer, 2001); and deontic modality involves speakers quantifying what they think is obligatory or necessary relative to a normative base, i.e. what they think *must* or *should* occur (Palmer, 2001). The 7 categories are as follows.

*The future tense* Sentences which use the future tense without any other modal words are classed in this category, e.g. *Tomorrow it will/is going to/shall rain* would be classed as future tense because it uses *will, is going to*, or *shall* and not also any modal class-criterion keywords (see below).

*The present tense* Sentences which are FTR but fail to be classed in any other category are classed as present tense, e.g. *tomorrow it rains* would be classed as present tense because it does not use the future tense or any other modal class-criterion keywords (see below).

*Low-certainty modality* Sentences which express low certainty about future events are classed in this category. This is further broken down depending on whether a modal verb or some other keyword class is used, e.g. *It could/may/might/should rain tomorrow* would all be classed as verbal-low-certainty because they use the low-certainty modal verbs *could, may, might* or *should*, while *It will possibly/probably/potentially rain tomorrow* and *I think it will rain tomorrow* would be classed as other-low-certainty because they express low certainty without using a modal verb. The reason for this subdivision is that our previous research, as well as review of the literature (Nuyts, 2000), suggests that modal verb use is more constrained by features of English grammar—modal verbs are obligatory whereas other-low-certainty constructions are not. We reasoned that individual differences in FTR usage would be more likely to manifest in the linguistic units which are freer to reflect speakers’ beliefs and intentions.

*High-certainty modality* Sentences which express high certainty are classed in this category, e.g. *It will definitely/certainty/absolutely rain tomorrow* would be classed as other-high-certainty. In English FTR, this domain is typically encoded using high-certainty epistemic adjectival or adverbial modifiers.

*Deontic modality* Sentences which express necessity and/or obligation are classed in this category, e.g. *You must come tomorrow* and *I have to/need to pick up more groceries tomorrow* would be classed as deontic modality because they use the deontic modal verb *must*, and deontic a *have to/need to* construction. These are typical ways of expressing deontic notions in English FTR (Nuyts, 2000).

*Bouletic modality* Sentences which express desires and hopes for the future are classed in this category, e.g. *I hope it rains tomorrow, I want it to rain tomorrow*, and *I wish it would rain tomorrow* would be classed as bouletic modality because they use the bouletic modal verbs *hope, want*, and *wish*.

*Irrealis* Sentences which express counterfactual realities are classed in this category, e.g. *If it rains, I’ll go out, If he would only go out with me, I would be happy* would be classed as irrealis because they express non-factual realities using the conditional *if*, and *would*.

Since the FTR-type classifier is intended to classify based on semantics, responses in the present tense which expressed modal semantics (e.g. *Tomorrow it could rain*) were classed according to the modal notions, and not formal tense categories. Similarly, responses in the future tense but which expressed modal notions (e.g. *Tomorrow it will possibly rain*) were classed according to the modal notions and not the tense category. This is because *It will possibly rain* and *It will rain* express different semantics and this is reflected in the classification scheme, see Fig. 1.

To check FTR-type classification accuracy on our Reddit data, we had trained assistants hand-annotate an evaluation dataset. To select data, we randomly selected from sentences for which $p_{{}_{FTR}}>=50\%$, n=50 from each FTR-type category, plus n=50 uncoded sentences, and n=50 randomly selected sentences (no overlaps). After exclusions for negations this resulted in N=447 sentences. We checked predictions against this evaluation dataset, and updated the FTR-type classification code to fix any discrepancies. In order to check this had not resulted in over-fitting to the evaluation dataset, we repeated this process with a second test dataset, this time randomly selecting n=30 sentences as described above (N=299 total sentences). Accuracy metrics against this test dataset are presented in Table 1.

### 2.2 Results

Descriptive statistics are presented in Table 2.

**Table 2:** Descriptive statistics for Studies 1 and 2.

study	variable	condition	n	$\bar{X}$	SD	min	max
Study 1	FTR	mental health	1,549,023	0.07	0.26	0	1
		control	465,158	0.06	0.25	0	1
	PTR	mental health	1,549,023	0.13	0.33	0	1
		control	465,158	0.09	0.29	0	1
	$\log_{e}\left(\right|H_{days}\left|\right)$	mental health	28,249	2.26	2.68	-8.97	11.39
		control	6999	2.77	2.91	-7.84	11.33
	$|H_{days}|$	mental health	28,249	332	2,134.62	0	88,850.2
		control	6999	923.93	4,629.42	0	83,386.52
	future tense	mental health	95,429	0.26	0.44	0	1
		control	25,302	0.27	0.45	0	1
	present tense	mental health	95,429	0.16	0.36	0	1
		control	25,302	0.17	0.37	0	1
	other-low-certainty	mental health	95,429	0.12	0.33	0	1
		control	25,302	0.1	0.3	0	1
	verbal-low-certainty	mental health	95,429	0.1	0.3	0	1
		control	25,302	0.09	0.28	0	1
	other-high-certainty	mental health	95,429	0.04	0.19	0	1
		control	25,302	0.04	0.2	0	1
	verbal-high-certainty	mental health	95,429	0.04	0.19	0	1
		control	25,302	0.03	0.18	0	1
	bouletic modality	mental health	95,429	0.17	0.38	0	1
		control	25,302	0.12	0.33	0	1
	irrealis	mental health	95,429	0.14	0.35	0	1
		control	25,302	0.14	0.34	0	1
Study 2	depression	-	202	6.86	9.66	0	40
	anxiety	-	202	4.4	7.07	0	34
	subjective temporal distance	-	2222	31.77	32.67	0	100
	$\log_{e}\left(k\right)$	-	202	-3.06	2.26	-9.06	1.74

Study 1 was intended to test the limited hypothesis that temporal horizons, frequency of time reference, and FTR-type habits would be different in the mental health condition as compared to control.

#### 2.2.1 Frequency of time reference

The first thing we established was how frequently redditors made non-present time reference. To address this, we estimated two regressions, one for past time reference and the other for future time reference. We used mixed regressions with a logistic link function and random intercepts clustered by redditor to regress the binary FTR and PTR variables resulting from Eq. (1) over *m.heal*, a dummy for control (0) versus mental health (1) condition:

(2) $$\log_{e}\left(\frac{\pi_{ij}}{1-\pi_{ij}}\right)=\beta_{0}+\beta_{1}m.heal_{i}+u_{j}$$

where $\pi_{ij}$ is the probability that $y_{FTR}=1$ for the future time regression, and $y_{PTR}=1$ for the past time regression, for observation i and redditor j. The random term $u_{j}$ allows intercepts to vary by redditor^4^ and is assumed to be drawn from the normal distribution with a mean of zero and a standard deviation drawn from the data, i.e. $u_{j}∼N\left(0,\sigma_{u}^{2}\right)$.

For mixed effects models like these, standard calculation of $R^{2}$ is complicated by the fact that there are multiple variance components explained by the model (i.e. fixed $\beta_{0}+\beta_{1}m.heal_{ij}$ and random $u_{j}$). We therefore used the *MuMIn* package (Bartoń, 2020) to calculate pseudo $R_{marginal}^{2}$ and $R_{conditional}^{2}$ (Nakagawa et al., 2017); $R_{marginal}^{2}$ represents the variance explained by the fixed effects, while $R_{conditional}^{2}$ represents the variance explained by the random effects + the fixed effects. For the FTR model, $R_{m}^{2}=0.001,\;R_{c}^{2}=0.019$ (where more than one method to calculate these quantities was available, we report the mean). This indicates that approximately 0.1% of the variance was explained by data-source condition. For the PTR model, $R_{m}^{2}=0.007,\;R_{c}^{2}=0.05$, indicating approximately 0.7% of variance was explained by data-source condition.

**Fig. 2:**
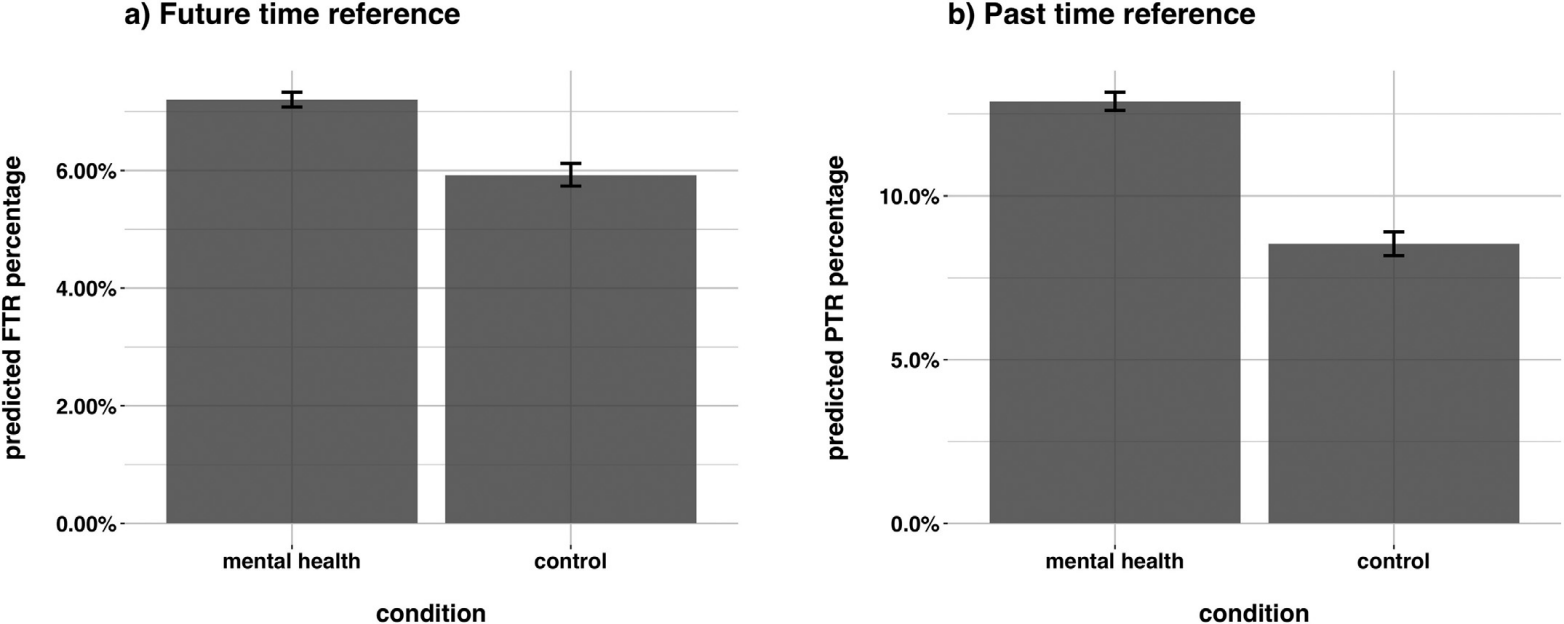
Differences in probability of making: (a) FTR and (b) PTR as a function of data-source condition in Study 1. Redditors in the mental health condition exhibited higher probability of referencing the past and future relative to control. Above and in Figs. 3 and 5, confidence intervals account for random effect variance by matrix-multiplying a predictor X by the parameter vector B to get the predictions, then extracting the variance-covariance matrix V of the parameters and computing XVX’ to get the variance-covariance matrix of the predictions. The square-root of the diagonal of this matrix represents the standard errors of the predictions, which are then multiplied by ±1.96 (Lüdecke, 2019).

We found that, relative to the control condition, redditors in the mental health condition were significantly more likely to make FTR, $e^{\beta }=1.23,\;SE=0.02,\;z=10.41,\;p<0.001$, and PTR, $e^{\beta }=1.58,\;SE=0.03,\;z=17.11,\;p<0.001$ (βs are exponentiated so represent changes in odds ratios). This indicates that redditors who had posted popular contributions to *r/Anxiety* and *r/depression* had approximately 23% higher odds of referencing the future and approximately 58% higher odds of referencing the past than redditors in the control condition, see Fig. 2.

**Fig. 3:**
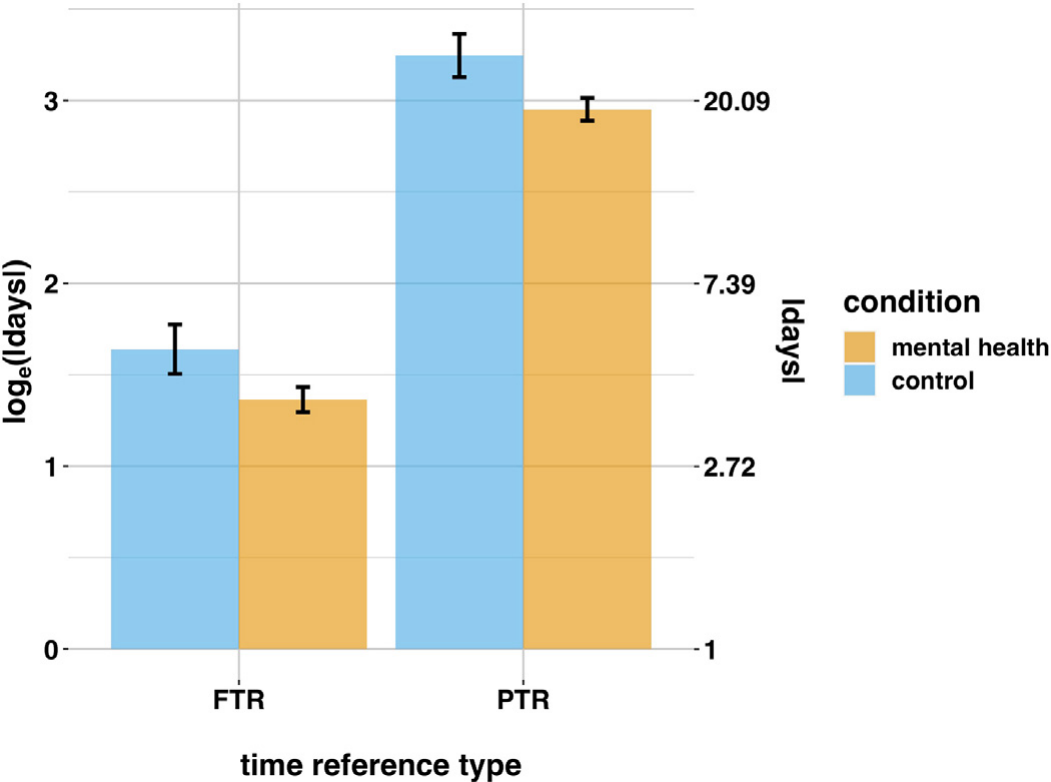
Differences in temporal horizons as a function of time reference type and data-source condition in Study 1. Redditors in the mental health condition exhibited contracted time horizons relative to control. Left *y*-axis is measured in the natural log of the absolute value of temporal distances (the dependent variable); right *y*-axis is the exponential transformation of the left *y*-axis, to yield the absolute value of time horizons (days).

#### 2.2.2 Future and past time horizons

Next we investigated whether there were differences in temporal horizons derived from SUTime. We excluded observations with no temporal expressions according to SUTime. Additionally, same-day past-time references were excluded. Recall, time horizons were calculated by subtracting the Reddit post date from the SUTime reference date. Inspection indicated that this procedure predominately resulted in false positives for same-day past-time references when SUTime used 00:00 h on the Reddit posting day as the reference date, e.g. *I’m seeing here tonight* posted at 16:00 3 August would result in a SUTime reference value of 00:00 3 August and a time delta of -16 h, even though it involves future time reference. Analyses were then conducted with the remaining n=35,248 observations. We created a dichotomous “time reference” dummy. For positive temporal horizons (i.e. FTR) this was 0; for negative temporal horizons (PTR) this was 1. We then transformed time horizons into a single, positive measure by taking their absolute value. The resultant temporal horizons were highly right skewed (skew=13.44), and truncated at zero. We therefore use the natural log of the absolute values of time horizon as our dependent variable. We regressed this over condition, the time reference dummy, and the interaction between them (in case effects of condition differed for future and past time horizons):

(3) $$\log_{e}\left(\right|H_{ij}\left|\right)=\beta_{0}+\beta_{1}m.heal_{i}+\beta_{2}t.ref_{i}+\beta_{3}m.heal_{i}t.ref_{i}+u_{j}+e_{i}$$

where t.ref is the time reference dummy; $u_{j}$ are random intercepts for participant, as above; and $e_{i}$ is an error term. Inspection of regression residuals indicated the log transformation resulted in a better approximation of regression assumptions of error normality than untransformed time horizons, so we proceeded. The fixed components of the model explained approximately 8% of the variance, $R_{m}^{2}=0.08,\;R_{c}^{2}=0.16$. We found redditors in the mental health condition had significantly shorter time horizons, $\left(e^{\beta }-1\right)\times 100=-24.42,\;SE=0.08,\;t\left(2791.38\right)=-3.56,\;p<0.001$ (βs are transformed as indicated so represent percentage change in y). This indicates that time horizons were approximately 24% shorter in the mental health condition, Fig. 3. There was also a significant effect of time reference. Compared with FTR, time horizons for PTR were significantly longer, $\left(e^{\beta }-1\right)\times 100=400.28,\;SE=0.07,\;t\left(35247.58\right)=24.69,\;p<0.001$. This indicates past time horizons were approximately 4 times longer than future ones, Fig. 3. The interaction term was not significant, $\left(e^{\beta }-1\right)\times 100=-1.98,\;SE=0.07,\;t\left(35242.89\right)=-0.27,\;p=0.786$. This indicates that there was no difference in the effect of data-source condition between PTR and FTR.

#### 2.2.3 FTR-type analysis

We next tested whether there were differences in how redditors referred to the future, i.e. in FTR type. We excluded all observations where $p_{{}_{FTR}}\leq 50\%$ according to the time reference classifier. This left n=145,130 observations. However, because of the keyword methods it employs, the FTR-type classifier cannot handle negations (e.g. *Rain tomorrow is not possible* expresses high certainty but would be classed as low-certainty because of the presence of the low-certainty keyword *possible*). We therefore detected the presence of negations use an averaged perceptron tagger following Collins (2002) but with Brown cluster features as described by Koo et al. (2008) and using greedy decoding (implemented in *spaCy* Explosion AI, 2020). We excluded any observations which used negations (n=24,399), leaving a final sample of n=120,731.

**Fig. 4:**
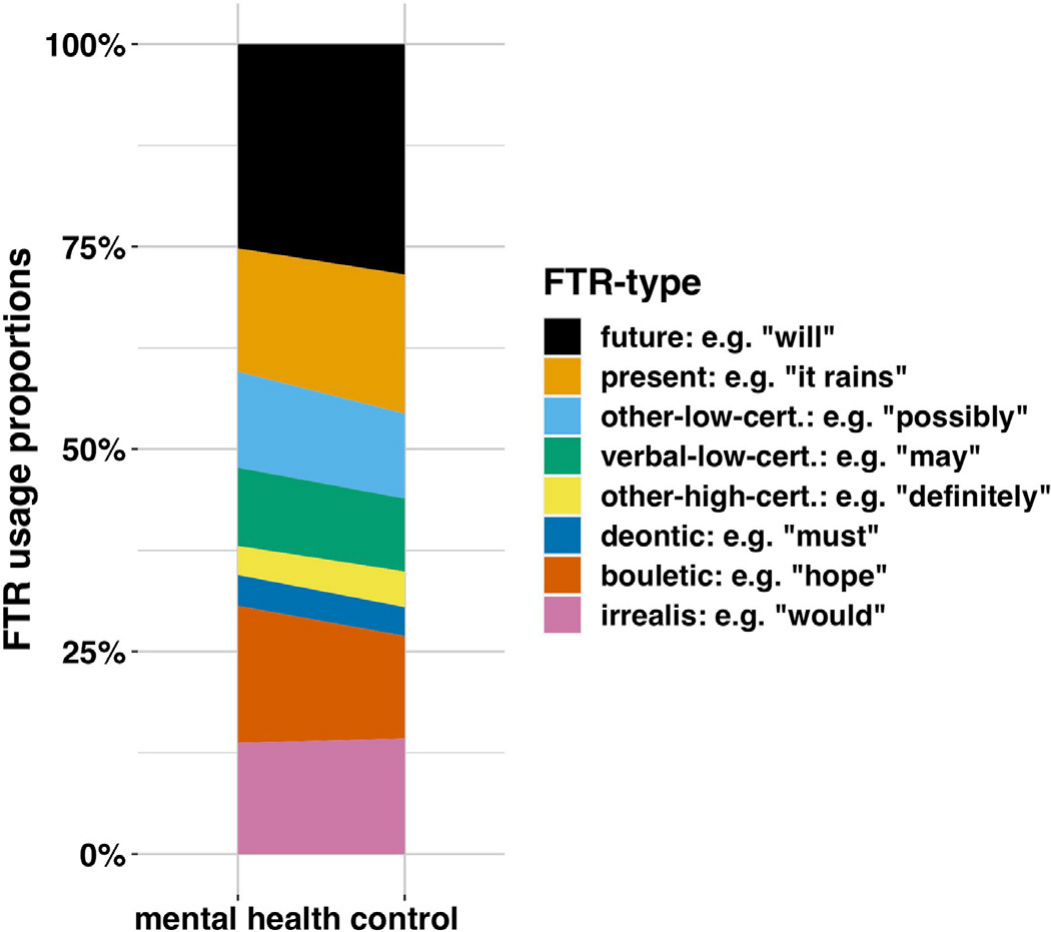
Plotted values are the proportional breakdown of FTR type in Study 1, by condition, i.e. the proportions per type normalised to sum to 1, $x_{k}=\left(\left(\sum_{i=1}^{n_{k}}x_{ik}\right)/n_{k}\right)/\sum_{i=1}^{k}x_{k}$ for k FTR types. This suggests FTR is spread across multiple semantic domains, not just tense (results are similar even when modality does not dominate tense [see SM]).

To give an overview, we present FTR-type usage proportions across the whole sample, Fig. 4. We found that, rather than the future tense marking the preponderance of FTR statements, FTR marking in English tends to be fairly equitably distributed across the different semantic categories estimated by the FTR-type classifier.

**Fig. 5:**
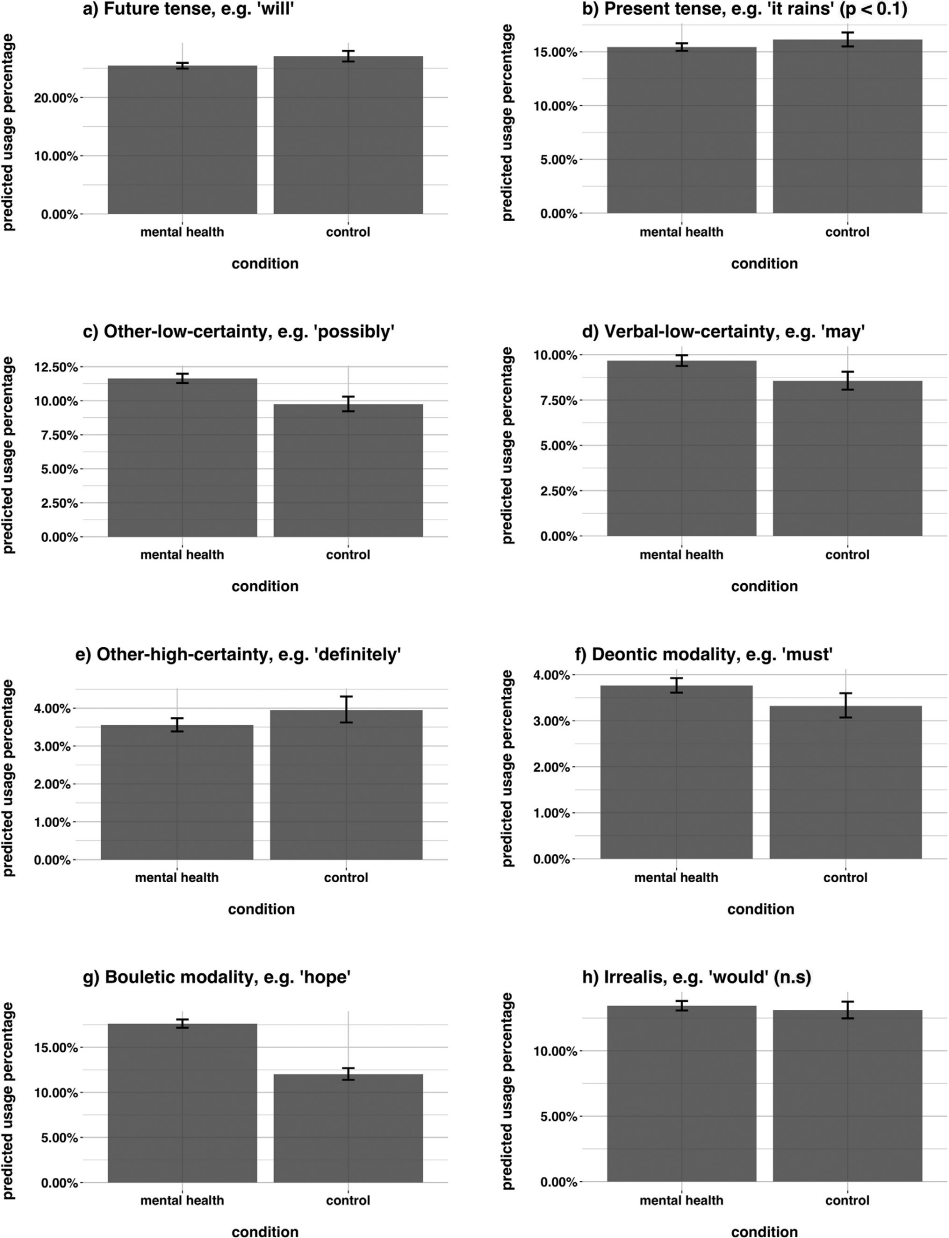
Differences in probability of using different FTR types when referring to the future as a function of data-source condition in Study 1. Redditors in the mental health condition exhibited higher probability of using low-certainty language, bouletic language, and verbal deontic language, and lower probability of using the future tense and other-high-certainty language.

To investigate whether there were differences between the control and mental health conditions, we regressed each FTR types over data-source condition with a logit link function and random intercepts clustered by redditor, i.e. Eq. (2) for each FTR-type variable. Statistical results are reported in Table 3 and depicted in Fig. 5. We found that redditors in the mental health condition were less likely to use the future tense, Fig. 5a, other-high-certainty constructions, Fig. 5e, and the present tense (for the present tense, this only approached significance, p=0.058)^5^, Fig. 5b. The semantic domain which all these FTR types encode is high certainty (Giannakidou, Mari, 2018, Salkie, 2010). This suggests that a bias against speaking about the future in high-certainty terms among sufferers of anxiety and depression may be driving this effect. This conclusion is supported by the fact that we found redditors in the mental health condition to be less likely to use low-certainty terminology (whether other or verbal, Fig. 5c–d). They also exhibited higher use of deontic modal terms, which encode future obligations and necessities, Fig. 5f. Additionally, redditors in the mental health condition used more bouletic modal language, which relates to hopes, plans, and intentions for the future, Fig. 5g. We found no difference in use of irrealis terms, Fig. 5h.

**Table 3:** FTR type by condition on Reddit (Study 1).

outcome	predictor	$e^{\beta }$	SE	z-score	
future tense	(Intercept)	0.37	0.02	-41.96	${}^{***}$
$R_{m}^{2}<0.001,\;R_{c}^{2}=0.023$	mental health condition	0.92	0.03	-3.11	${}^{**}$
present tense	(Intercept)	0.19	0.02	-67.42	${}^{***}$
$R_{m}^{2}<0.001,\;R_{c}^{2}=0.016$	mental health condition	0.95	0.03	-1.89	·
other-low-certainty	(Intercept)	0.11	0.03	-70.7	${}^{***}$
$R_{m}^{2}=0.001,\;R_{c}^{2}=0.028$	mental health condition	1.22	0.04	5.56	${}^{***}$
verbal-low-certainty	(Intercept)	0.09	0.03	-74.01	${}^{***}$
$R_{m}^{2}<0.001,\;R_{c}^{2}=0.025$	mental health condition	1.14	0.04	3.71	${}^{***}$
other-high-certainty	(Intercept)	0.04	0.05	-69.4	${}^{***}$
$R_{m}^{2}<0.001,\;R_{c}^{2}=0.042$	mental health condition	0.9	0.05	-2.07	${}^{*}$
deontic	(Intercept)	0.03	0.04	-80.02	${}^{***}$
$R_{m}^{2}<0.001,\;R_{c}^{2}=0.021$	mental health condition	1.14	0.05	2.71	${}^{**}$
bouletic modality	(Intercept)	0.14	0.03	-63.61	${}^{***}$
$R_{m}^{2}=0.007,\;R_{c}^{2}=0.041$	mental health condition	1.57	0.04	12.72	${}^{***}$
irrealis	(Intercept)	0.15	0.03	-65.91	${}^{***}$
$R_{m}^{2}<0.001,\;R_{c}^{2}=0.025$	mental health condition	1.03	0.03	0.88	

Coefficients represent the change in odds ratio of each predictor compared to the intercept. ${}^{***}p<0.001$; ${}^{**}p<0.01$; ${}^{*}p<0.05$; ·p<0.1.

### 2.3 Discussion

Taken together, the Study 1 results present an interesting pattern. We found that when making FTR, redditors in the mental health condition were more likely to use low-certainty terminology, bouletic terminology, and deontic terminology, and less likely to use the future tense, the present tense (p=0.0.58), and high-certainty terminology. The future tense (Giannakidou, Mari, 2018, Salkie, 2010), the present tense (Giannakidou, Mari, 2018, Salkie, 2010), and other-high-certainty terms all encode notions of modal high certainty. The finding that redditors in the mental health condition used more of these high-certainty FTR types, and fewer low-certainty FTR types, suggests that the experience of anxiety and/or depression may be characterised by increased uncertainty about future events. Additionally, higher use of bouletic terminology suggests increased desire for future change.

We found redditors in the mental health condition were more likely to make past and future time reference and their time horizons were shortened relative to control. This suggests that anxiety and depression may be characterised by heightened salience of future and past events, but within a shorter temporal window.

We found no differences between the anxiety and depression conditions. We reasoned that self-selecting to post in *r/Anxiety* and *r/depression* was a weak diagnostic criteria, especially given high co-morbidity rates between the two mood disorders (Groen et al., 2020). We therefore undertook to differentiate the relationships between anxiety, depression, and temporal horizons in a survey paradigm over which we could exert a greater degree of control.

## 3 Study 2

Our goals for Study 2 were twofold. First, we wanted to understand more about representations of temporal distance and how this might relate to temporal discounting in sufferers of anxiety and depression. We hypothesised that shortened temporal horizons in sufferers of anxiety and/or depression might be driven by temporally distal representations future events. If sufferers of anxiety and depression tend to talk about temporally proximal time frames on Reddit, this might be because they represent such dates as subjectively more distal. Distal representations of subjective temporal distance is a predictor of increased temporal discounting (Kim, Zauberman, 2009, Thorstad, Nie, Wolff, 2015, Zauberman, Kim, Malkoc, Bettman, 2009). This means such perceptual biases might be driving increased temporal discounting in sufferers of anxiety. We therefore hypothesised that sufferers of anxiety and/or depression would represent future events more distally than healthy individuals, which would in turn give rise to increased temporal discounting. Second, we aimed to disambiguate whether such cognitive biases were characteristic of anxiety, depression, or both.

### 3.1 Materials and methods

#### 3.1.1 Participants

A sample of N=202 participants (n=103 females, n=98 males, and n=1 other) passed attention checks to complete the study (see Section 3.1.2). Data were collected in March 2020. All participants were English speakers residing in the United Kingdom and were at least 18 years old. Participants were recruited from Amazon Mechanical Turk Prime and completed the survey online. The study was approved by the internal review board of Brunel University London (Ref. 22348-MHR-Jan/2020- 24407-1). Participants were paid the UK living wage on a pro-rata basis.

#### 3.1.2 Materials

Study 2 consisted of 3 tasks: (1) an intertemporal-choice task designed to establish temporal discounting; (2) a “time-slider” task designed to establish subjective representations of future temporal distance; and (3) the Depression Anxiety and Stress Scales 21 item measure (DASS-21) which measures depression, anxiety, and stress levels (Lovibond and Lovibond, 1995).

*The intertemporal-choice task* In this task, participants made repeated binary decisions between a larger reward (the “larger-later reward”) offered after a delay and a smaller reward (the “smaller-sooner reward”) offered immediately; for instance, “Would you prefer £50 now of £100 in six months?” The smaller-sooner reward was always smaller than the larger-later reward, which was fixed at £100. The factors of this task were the amounts of the smaller-sooner reward (£50–£95 by increments of £5), and the delays of the larger-later reward (later today, tomorrow, one week, one month, two months, three months, six months, one year, two years, five years, and ten years). If participants chose the larger-later reward, this was scored with (1), otherwise (0). Amounts and delays were fully crossed to produce a test battery of $10_{amounts}\times 11_{delays}=110_{items}$. Prior to starting, participants were told to “Try to answer quickly and intuitively, without thinking about it too much.” Item order was randomised and one item was displayed per page.

The following attention check was implemented. At two random points during the task, participants were given a choice between two present-time rewards of different values. If they chose the smaller reward, they were ejected from the survey immediately.

To be able to model linear relationships between discounting (which is non-linear over time Green and Myerson, 2004) and participant-level variables (anxiety, depression), we calculated a participant-level measure of discounting. Research has shown that the following hyperbolic function fits real and hypothetical delay discounted value well (Kirby, Petry, Bickel, 1999, Mazur, 1987):

(4) $$V=\frac{A}{1+kD}$$

where V is the subjective value of a delayed reward A at a given delay D, and k is a scaling parameter which captures individual differences in discounting. Higher values of k imply lower V, i.e. more discounting. To derive participant-level ks, we followed Kirby et al. (1999). This involved calculating hypothetical values of k and retaining for each participant some k which best predicted empirical choices. Specifically, we calculated all $k_{1-n}$ at indifference between LLR and SSR for each D and SSR, i.e. all ks such that $SSR=LLR/(1+k_{i}D)$ for all values of SSR and D under the study. We then predicted hypothetical intertemporal choices for each $k_{1-n}$ and retained $k_{i}$ for each participant $p_{j}$ which had the highest proportion of matches against $p_{j}$ empirical choices. When more than one k had an equal number of matches, we took the geometric mean (Kirby et al., 1999). The resultant k values correctly predicted participants empirical choices for 93.5% of observations. Since k tends to be non-normally distributed, we used $\log_{e}\left(k\right)$ as our participant-level measure of discounting (Kirby et al., 1999). This is the dependent variable for this task.

*The time-slider task* In this task, participants used a slider to rate whether they construed a given objective temporal distance between “close to now” (0) and “far from now” (100). To increase a sense of subjectivity, numbered slider intervals were not displayed. This was based on similar methods in Zauberman et al. (2009) and Kim and Zauberman (2009). Rather than time horizons—how far into the future participants tend to think—the time-slider task established how subjectively distant participants felt given temporal distances to be. The only factor was the objective temporal distances. These matched the intertemporal-choice task. For each item, participants were directed to “indicate with the slider how far away from NOW the given time feels to you.” Prior to starting, participants were given a training example involving a past time reference (9 months ago), and were told to “try to answer quickly and intuitively, without thinking about it too much.” Item order was randomised and one item was displayed per page.

*The Depression, Anxiety, and Stress Scale* To establish depression and anxiety levels, participants completed DASS-21 (Lovibond and Lovibond, 1995). In the DASS-21, participants self-reported using a Likert scale what extent a given statement applied to them. The Likert scale options were “did not apply to me at all” (0), “applied to me to some degree, or some of the time” (1), “applied to me to a considerable degree, or a good part of time” (2), and “applied to me very much or most of the time” (3). For each item, participants were instructed to “Please read each statement and use the scale provided to indicate how much the statement applied to you over THE PAST WEEK. There are no right or wrong answers. Do not spend too much time on any statement.” The DASS-21 is broken down into 3 dimensions, each comprising 7 items, designed to separately measure depression, anxiety, and stress. Example items from each dimension are as follows: “I felt I wasn’t worth much as a person” (depression), “I felt I was close to panic” (anxiety), and “I found it difficult to relax” (stress). See SM for items. The DASS-21 is not a diagnostic tool *vis-à-vis* the discrete diagnostic categories of, for instance, the Diagnostic and Statistical Manual of Mental Disorders (American Psychiatric Association, 2013). Rather, it is based on research which suggests that the differences in depression, anxiety, and stress experienced by healthy and clinical populations are a matter of degree (Lovibond and Lovibond, 1995). It is therefore designed to span the boundaries between clinical and healthy populations. Dependent variables are the sum of scores for each dimension multiplied by 2, and therefore theoretically range between (0) and (42).

#### 3.1.3 Procedure

The survey was hosted on Qualtrics. It had a within-subjects correlational design, meaning there were no independent variables or factors other than the within-subjects factors described above for each task. Participants provided informed consent; indicated if they were male, female, or other; confirmed they were 18 or over; and confirmed they were native English speakers. Participants who indicated they were under 18 or not native English speakers were ejected from the survey immediately. Participants completed the intertemporal-choice task, the time-slider task, and then the DASS-21.

### 3.2 Results

**Fig. 6:**
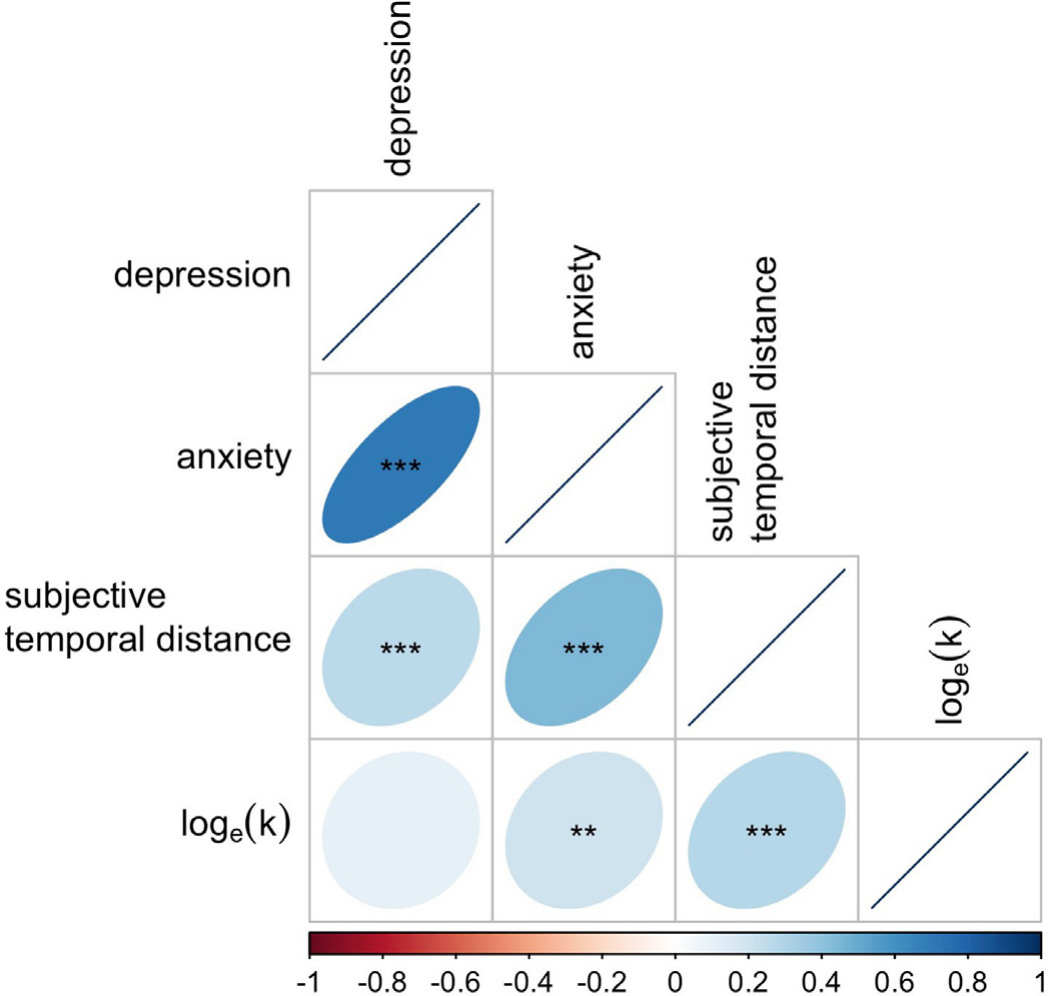
Bivariate Pearson correlations for Study 2. ${}^{***}p<0.001$; ${}^{**}p<0.01$; ${}^{*}p<0.05$; ·p<0.1.

Descriptive statistics for Study 2 are presented in Table 2. Correlations are presented in Fig. 6. Anxiety and depression scores were positively correlated, which may reflect widely noted co-morbidity between the two mood disorders (Groen et al., 2020). Both anxiety and depression scores were positively correlated with subjective-temporal-distance ratings. This suggests sufferers of anxiety and depression represent future events more distally. Subjective delay ratings were positively correlated with $\log_{e}\left(k\right)$ scores. This indicates that participants who represented future outcomes as more distal also exhibited increased temporal discounting.

To understand the conditional relationship of anxiety and depression on discounting via temporal-distance ratings, we conducted a mediation analysis. In mediation analyses, a predictor X is assumed to effect an outcome Y via a mediating variable M. For instance, vitamin C levels mediate the relationship between eating citrus fruit and the development of scurvy (Pearl and Mackenzie, 2018). Mediation analyses can be conducted by combining two regressions into a single model: an X→M regression and as X+M→Y regression. In mediation terminology, the X→M path is refereed to as α, the M→Y path is referred to as β, and the X→Y path is referred to as $\tau^{\prime }$. The “indirect effect”, X→M→Y, can be estimated as the product of the paths involved, αβ, and captures the effect of X on Y via M while controlling for the “direct effect” $\tau^{\prime }$. Similarly, the direct effect captures the effect of X on Y while controlling for the indirect effect. The “total effect” is the sum of αβ and $\tau^{\prime }$, and captures the total effect of X on Y, as in a normal regression (Yuan and MacKinnon, 2009).

**Fig. 7:**
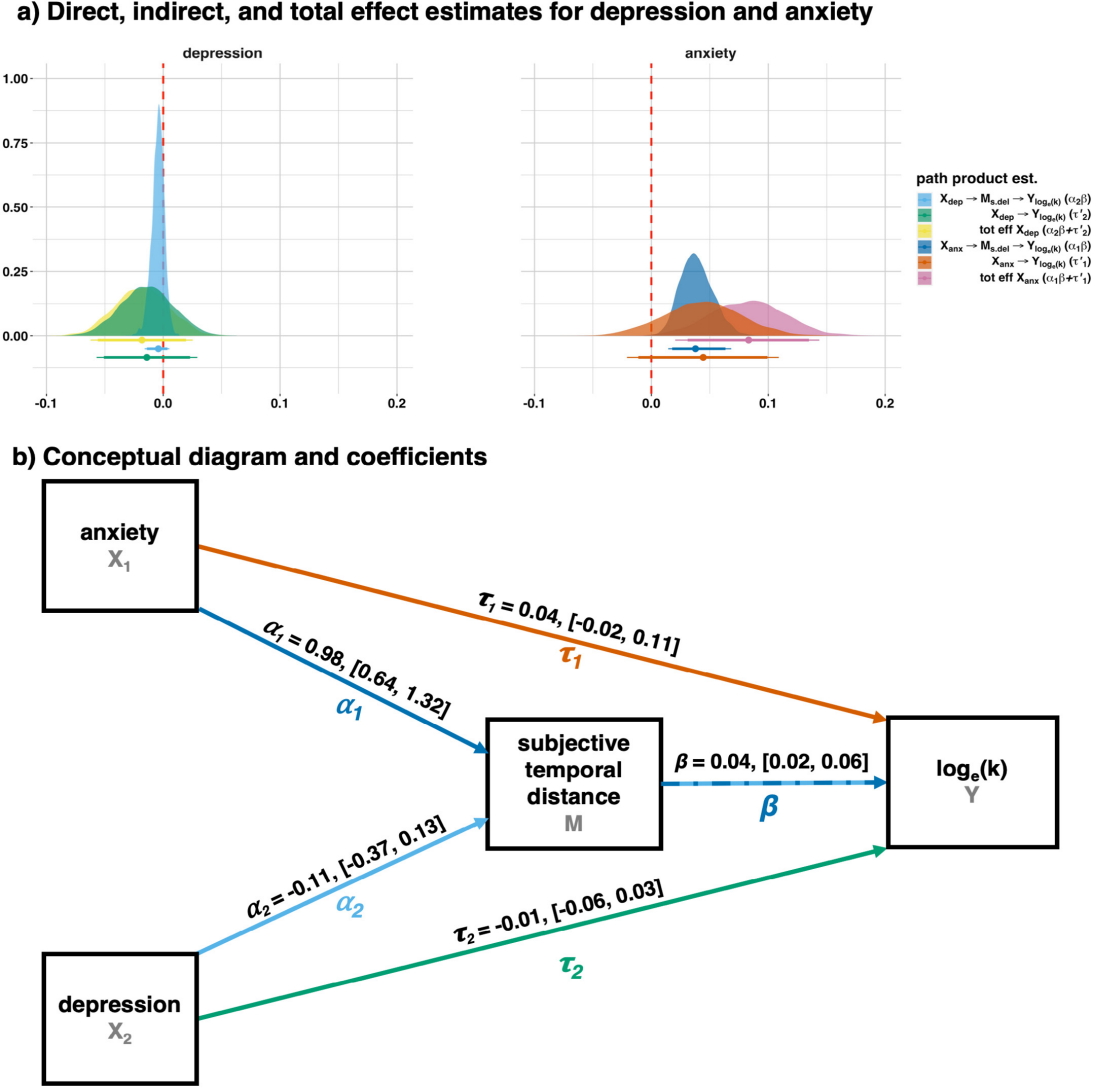
Study 2 mediation results. a) Posterior estimates for paths and path products for the effects of anxiety and depression on discounting ($\log_{e}\left(k\right)$) via participant-level mean ratings of subjective future temporal distance. Bayesian Credibility Intervals (CI) are plotted at 90% (thick bars) and 95% (thin bars) probability; these are the domain of the posterior probability distribution that approximates the parameter at the given probabilities. For two-tailed hypotheses, thin bars represent cut-off criteria. For one-tailed hypotheses, thick bars do (providing sign is as predicted). b) Conceptual diagram, with parameter point estimates and 95% CIs. If the CI does not contain zero, parameters are significantly different from zero. The values for the intercepts are, $\lambda_{1:subj.dist.}=28.25,\;\left[26.10,30.42\right]$, and $\lambda_{2:\log_{e}\left(k\right)}=-4.41,\left[-5.16,-3.64\right]$.

We used $\log_{e}\left(k\right)$ as our outcome variable (Y), anxiety and depression scores from the DASS-21 as our predictor variables ($X_{1,2}$), and participant-level mean subjective temporal-distance scores as our mediating variable (M). The model therefore took the following form:

(5) $$\begin{array}{cc} subj.dist_{i} & =\lambda_{1}+\alpha_{1}anx_{i}+\alpha_{2}dep_{i}+e_{1i}\\ \log_{e}{\left(k\right)}_{i} & =\lambda_{2}+\tau_{1}^{\prime }anx_{i}+\tau_{2}^{\prime }dep_{i}+\beta subj.dist_{i}+e_{2i} \end{array}$$

where $\lambda_{1,2}$ are intercepts, $\alpha_{1,2}$ are slope coefficients for the effects of anxiety and depression on mean subjective temporal distance, $\tau_{1,2}^{\prime }$ and β are slope coefficients for the effects of anxiety, depression, and mean subjective distance on $\log_{e}\left(k\right)$ temporal discounting, and $e_{1,2}$ are error terms. See Fig. 7b for conceptual diagram.

Bayesian statistics are well-suited to mediation analyses. As well as making no assumptions about sampling statistic normality, they allow for straight-forward inferences about any transformation of model parameters (i.e. path products) through simply carrying out the desired operation on posterior probability distributions (Vehtari et al., 2019). We therefore used the *brms* package (Bürkner, 2017) to derive posterior probability distributions for model parameters using a no U-Turn Hamiltonian Monte Carlo sampling procedure (Hoffman, Gelman, 2014, Stan Development Team) with uninformative priors (i.e. Unif(−∞−∞)), 4 chains, 2,000 iterations, and a burn in discard of 1,000 (the defaults). Inspection of caterpillar plots and $\hat{R}$s indicated that the estimation procedure had converged (all $\hat{R}s==1.00$ at convergence).

The predictors explained moderate proportions of the variance, $R_{subj.temp.dist}^{2}=0.2,\;CI_{95\%}=\left[0.11,0.29\right]$, $R_{\log_{e}\left(k\right)}^{2}=0.1,\;CI_{95\%}=\left[0.04,0.18\right]$. These are a Bayesian adaptation of $R^{2}$ following (Gelman et al., 2018), including spread statistics. They indicate that anxiety and depression explained approximately 20% of the variance in subjective temporal distance; and anxiety, depression, and subjective temporal distance explained approximately 10% of the variance in temporal discounting ($\log_{e}\left(k\right)$ scores).

Results are reported in Fig. 7. We had made the directional hypotheses that anxiety and/or depression would be associated with more distal subjective distances and therefore increased discounting—higher $\log_{e}\left(k\right)$. We tested the one-tailed hypotheses that these effects would be positive. As we had predicted for anxiety, we found a significant positive indirect effect, $Est.=0.04,\;CI_{90\%}=\left[0.02,0.06\right],\;pp>0.999$^6^, and total effect, $Est.=0.08,\;CI_{90\%}=\left[0.03,0.14\right],\;pp=0.998$, see Fig. 7a. The direct effect of anxiety was not significant, $Est.=0.04,\;CI_{90\%}=\left[-0.01,0.1\right],\;pp=0.914$. This indicates that participants who self-reported as high-anxiety also had more distal representations of future events. As a consequence, they devalued future outcomes to a greater extent. The non-significant direct effect indicates that the entirety of the effect of anxiety on temporal discounting was transmitted via construals of temporal distance, Fig. 7.

On the other hand, we found no significant effects for depression (indirect: $Est.=0,\;CI_{90\%}=\left[-0.01,0\right],\;pp=0.194$, direct: $Est.=-0.01,\;CI_{90\%}=\left[-0.05,0.02\right],\;pp=0.265$, total: $Est.=-0.02,\;CI_{90\%}=\left[-0.06,0.02\right],\;pp=0.207$, see Fig. 7a). see Fig. 7a). This indicates that, when controlling for anxiety, depression scores had no impact on temporal distance or temporal discounting, Fig. 7, despite bi-variate correlations between depression and temporal-distance ratings, Fig. 6.

It is possible that a random question-answering strategy by some participants drove the results. Specifically, if anxious and/or depressed participants were more likely to answer the intertemporal choice task questions randomly, this could have affected values of *k* (Franco-Watkins et al., 2006). The proportion of future choices across the experiment was.44. If depressed and/or anxious participant were more likely to answer randomly, this would have increased to.5 in correspondence with DASS scores. This would have artificially increased *k* values (Franco-Watkins et al., 2006). Conversely, if anxious and/or depressed partcicipants were less likely to answer randomly, this could have lowered *k*. To test this, we re-estimated the mediation model including a measure of choice stochasticity: The proportion of choices each participant made which disagreed with the predicted choice under the optimal value of *k* (Franco-Watkins et al., 2006). This did not qualitatively change reported results, or significantly predict any differences in $\log_{e}\left(k\right)$. This suggests a random-answering strategy by anxious and/or depressed participants did not drive results (SM).

### 3.3 Discussion

An important consideration is the reliability of the measures. The test-retest reliability of the time slider task is not well known, but it appears to have good construct and concurrent validity (Bradford, Dolan, Galizzi, 2019, Kim, Zauberman, 2009, Zauberman, Kim, Malkoc, Bettman, 2009). Temporal discounting has well established test-retest reliability (Anokhin, Golosheykin, Mulligan, 2015, Baker, Johnson, Bickel, 2003, Beck, Triplett, 2009, Johnson, Bickel, Baker, 2007, Kirby, 2009, Ohmura, Takahashi, Kitamura, Wehr, 2006, Simpson, Vuchinich, 2000, Smits, Stein, Johnson, Odum, Madden, 2013, Weafer, Baggott, de Wit, 2013). Similarly, the DASS shows good test-retest reliability, as well as construct validity and factor structure (da Silva, Passos, de Oliveira, Palmeira, Pitangui, de Araújo, 2016, Gomez, Summers, Summers, Wolf, Summers, 2014, Wang, Shi, Geng, Zou, Tan, Wang, Neumann, Shum, Chan, 2016). This suggests findings can be interpreted as valid, though caution is prudent in light of the unknown test-retest reliability of the time-slider task.

Caveats notwithstanding, the findings suggest that anxious participants tended to construe future events more distally, and that this in turn caused them to temporally discount to a greater degree. The fact that the direct effect of anxiety was not significant indicates the entirety of the effect of anxiety on temporal discounting was mediated by subjective-temporal-distance ratings. This suggests reported associations between anxiety and temporal discounting are mediated by construals of future temporal distance. On the other hand, we found no significant relationships between depression and subjective temporal distance or temporal discounting.

## 4 General discussion

In Study 1, we found that language used by redditors who had posted to *r/Anxiety* and *r/depression* differed significantly from control. Firstly, we found they were more likely to make non-present time reference, both past and future. This suggests that sufferers of anxiety and/or depression may spend more time thinking about future and past events rather than present ones. It may be important to note that we did not build a tri-class past/present/future time reference classifier, so the reference category in these analyses is not present time. Rather it is anything that is not future or past time reference, for instance statements of ability *It can rain a lot here*; statements of fact, *The sun is hot*; or present time reference, *I am hot*. In light of this, it would be incorrect to infer that redditors in the mental health condition spent less time making present time reference. Rather, they spent more time making past and future time reference. An important point is that this finding does not pertain to language use differences between *r/Anxiety* and *r/depression* on the one hand and *r/All* on the other. The snowball data-collection method meant that the mental health condition comprised post made from across Reddit by redditors who had posted “hot” comments to *r/Anxiety* and *r/depression* on the day we downloaded the data. Language use differences are therefore plausibly attributable to psychological traits shared by people who suffer with these mood disorders, at least insofar as posting to online forums is a reliable diagnostic criteria.

While relationships between future time perspective and anxiety and depression have been established, such research does not illuminate the extent to which the future is salient to sufferers from these mood disorders. As far as we know, this is the first study to find that the experience of anxiety and depression may be characterised by an increased amount of time spent talking about the future and the past.

We also found significant (though small) differences in FTR type. This suggests that habits of thinking about the future may differ between healthy populations and sufferers of anxiety and/or depression. The largest differences seemed to be driven by lower use of the future tense and higher use of bouletic language relative to control, Fig. 5. The semantic domain which bouletic modal language encodes is desiderative, having to do with hopes, desires, and plans for change. This suggests that future thinking in sufferers of anxiety and/or depression is characterised by desire for future change rather than simply statements of fact.

We found higher use of low-certainty language relative to control. This suggests that sufferers of anxiety and/or depression may construe the future more uncertainly then healthy populations. This raises the possibility that probability discounting may be a relevant marker of anxiety and/or depression. Probability discounting is similar to delay discounting, but involves the devaluation of risky rather than delayed rewards (Green and Myerson, 2004). It is measured in a similar way, by giving participants binary choices, but rather than immediate and delayed, the choice is between certain and risky rewards; for instance a 100% chance of receiving $10 vs. a 50% chance of receiving $20. People who probabilistically discount more would tend to choose high-certainty options because they devalue low-certainty ones to a greater degree. Recent research involving rewards which are both probabilistic and delayed, e.g. a 100% chance of receiving $10 vs. a 50% chance of receiving $20 in three months, has found that these factors interactively predict discounting rates (Vanderveldt et al., 2015). This suggests that real-world intertemporal decision making (in which people must balance present vs. future options as in delay discounting paradigms) which usually involves some degree of uncertainty about the future may be driven by both probabilistic and temporal discounting rates.

The relationship between probability discounting and anxiety and/or depression is understudied relative to temporal discounting. While one study found probability discounting was not a significant marker of major depressive disorder (Hart et al., 2019), our results suggest that anxiety and/or depression may be characterised by low-certainty construals of future events. This may warrant further investigation into potential relationships between anxiety, depression, and low-certainty future construals and their role in risky intertemporal decision-making processes.

Additionally, we found higher use of deontic modal language relative to control. The primary deontic modal verb in English is *must*, and the other criterion keywords in the FTR type classifier in this category are *have to, need to*, and variants thereof. This suggests that redditors in the mental health condition were more likely to encode *obligation* and *necessity*. This echoes the finding that people who experience mental health distress overuse “absolute words”. Specifically, words with totalising predication, for instance *absolutely, always, completely, constant, definitely, entire, every, everyone, everything, full, must, never, nothing, totally* and *whole* (Al-Mosaiwi and Johnstone, 2018). Though these words encode more than deontic modal language, there is some overlap between the predicate function of these lexical expressions of absoluteness, and the totalising nature of strong modal expressions of obligation (*need to, have to, must*) and conditional necessity (*If X, must Y*).

It is unclear whether these patterns are causal or descriptive. In other words, habitually construing future events in low-certainty, highly-constrained, bouletic terms may contribute to the development and maintenance of anxiety and/or depression. On the other hand, such FTR usage differences may simply be symptomatic of anxiety and/or depression. In either case, we appear to have isolated new markers of these mood disorders vis-à-vis FTR.

Study 1 found contracted time horizons in the mental health condition relative to control. In other words, redditors in the mental health condition referenced more proximal future and past times. In Study 2, we used a different measure of subjective temporal distance. Grasping this difference is crucial. The time horizon measured in Study 1 approximated how far into the future people tended to talk about. The subjective-temporal-distance measure in Study 2 gave participants objective future dates and asked them how far away they construed those dates to be. We found that anxious participants tended to rate future events as more temporally distal. This suggests that subjective representations of temporal distance (i.e. how far away DATE feels to an individual), and time horizons (i.e. how far into the future an individuals tends to think) may be negatively correlated. More research using time slider tasks and the Wallace task could explore this further.

In Study 2, we found subjective-temporal-distance ratings mediated a relationship between anxiety and temporal discounting. This suggests that subjective representations of future time may be partially driving reported links between anxiety and temporal discounting (Rounds et al., 2007), and adds evidence to the inference that the relationship is significant and positive (contra Jenks, Lawyer, 2015, Steinglass, Lempert, Choo, Kimeldorf, Wall, Walsh, Fyer, Schneier, Simpson, 2017). On the other hand, we found no relationship between our measures of discounting and depression, despite the fact that depression has been previously linked to contracted future time horizons (Dilling and Rabin, 1967), and increased temporal discounting (Felton, Strutz, McCauley, Poland, Barnhart, Lejuez, 2020, Pulcu, Trotter, Thomas, McFarquhar, Juhasz, Sahakian, Deakin, Zahn, Anderson, Elliott, 2014). This may be driven by symptom severity and/or reward size effects. Reward size has a well-established effect on discounting rates; larger future rewards are discounted less, i.e. people are more willing to wait when future rewards are large even when present rewards are proportionally identical (Green and Myerson, 2004). Different reward sizes may therefore be responsible for differences in reported findings. Further, there is evidence that reward size may affect temporal discounting in depressive individuals differently to healthy ones. Pulcu et al. (2014) compared discounting between clinically diagnosed present sufferers of Major Depressive Disorder (MDD), remitted MMD (rMDD), and healthy controls, with small (£30), medium (£55) and large (£80) rewards. They found no significant differences between rMDD participants and controls, and only found significant differences between MDD and controls for large rewards. Since we used a reward size comparable to their large condition (£100), this suggests that depressive symptom severity in our sample was not high enough to detect an effect. It is an important point that depression was significantly correlated with subjective-temporal-distance scores, Fig. 6. This suggests that—had we not also measured anxiety—depression would have had an impact on temporal discounting via subjective temporal distance. However, when anxiety was also included as a predictor in the mediation analysis, there was no significant effect of depression Fig. 7. Since the two mood disorders are often co-morbid (Groen et al., 2020), our results suggest including measures of both is important when attempting to understand the relationship between either and time perspective.

### 4.1 A functionalist perspective on the results

Functionalist approaches to mental health and wellness offer a theoretical framework which may help make sense of our findings. A functionalist perspective simply asks “what is the purpose of a mental state?” The relevant mental states here are those associated with anxiety and depression. Purpose is understood in terms of the relationship between an organism and its environment. In this view, mental states and their suites of behavioural correlates are seen to help organisms survive in stochastic but statistically predictable environments (see Brunswik, 1952, Brunswik, 1956). A functionalist approach to the understanding and treatment of mental health therefore assumes there is a utility (a function) to common patterns of thought and feeling–even those which seem exaggerated in people who suffer from mental health problems.

This is contrasted with the descriptive approach taken in, for instance, the DSM-5, which seeks to describe the commonly-occurring attributes of the various mental illnesses (American Psychiatric Association, 2013). From the taxonomic perspective of the DSM-5, anxiety and depression look profoundly different. In anxious states, individuals experience heightened autonomic arousal (Rozenman et al., 2017). Systolic blood pressure may rise (Gerra et al., 2000), and heart rate may rise (Rozenman et al., 2017), or exhibit suppressed variability (Rozenman et al., 2017), and breathing rate may be more variable (Wilhelm et al., 2001). Patients tend to describe recursive loops of rapid negative thoughts, or constant ideation of potential catastrophes. Emotions may be dysregulated, with powerful experiences of fright and frustration often occurring. The mind and body are poised for action, and are vigilant for potential dangers. The phenomenology of depressive states is markedly different. Individuals experience reduced autonomic arousal (Volkers et al., 2003). Endogenous opioids (endorphins) rise (Al-Fadhel et al., 2019), and motor activity is reduced (Volkers et al., 2003). Rather than a racing mind, patients report their thinking becomes slow and foggy. Emotions feel dulled, if not completely numbed. Physical energy is low, and patients describe feeling as though they are in a state of lassitude and torpor.

Despite these marked differences, a functionalist approach takes the view that anxiety and depression are similar because they are both strategies which sufferers employ to deal with perceived “threats” to their safety and/or well-being. A useful way of understanding what is meant by “threat” is the concept of homeostatic salience. This refers to elements of the environment which are experienced as relevant to immediate well-being (Suvak and Barrett, 2011). For instance, ensuring the provision of one’s basic physical needs has clear homeostatic relevance. Even in a simple physical example, there are individual differences in what may be construed as a threat. Someone living in an air-conditioned building connected to a robust power grid would be unaffected by an approaching heat wave, but the same phenomenon would have high homeostatic salience to someone living without such amenities. However, modern approaches to clinical psychology understand more complicated feelings of belonging and attachment can also have high homeostatic salience (at least since Bowlby, 1969). In this view, there are a vast array of ways people feel they might be harmed. Clinicians working from a functionalist perspective are therefore inclined to see anxiety and depression as useful, if different, strategies for dealing with threats to homeostatic regulation. Put another way, functional clinicians try to understand the, possibly idiosyncratic, ways in which sufferers of anxiety and depression construe their environment as threatening. This implies that in most cases of anxiety and depression, clinicians can expect to find something which the sufferer perceives as having homeostatic salience.

This line of thinking clarifies the present results in several ways. First, redditors in the mental health condition used more bouletic FTR language, which involves the expression of desire and intention. This suggests a neutral, objective, or uninvested viewpoint may be inaccessible. If anxiety and depression are coping strategies to deal with homeostatic threats, it makes sense that sufferers would be more invested in future outcomes. They would therefore use more language which expresses desire for specific eventualities. Secondly, redditors in the mental health condition exhibited contracted time horizons relative to controls. In threatening environments, distal time frames lose salience. To someone who is hypothermic, nothing is important relative to the immediate provision of warmth. The need to get warm constricts which possibilities matter, as well as the timeline on which they matter. In navigating an environment which is construed as threatening, sufferers of anxiety and depression may disregard distal events which seem irrelevant compared with more proximal ones. Third, redditors in the mental health condition used more low-certainty FTR constructions. Uncertainty and threat are closely related. Mild hypothermia may be disregarded if a warm house is minutes away. On the other hand, uncertainty about environmental affordances seems inherently threatening. Increased low-certainty language use may therefore either: (a) reflect threatening construals about which sufferers are uncertain; or (b) contribute to low-certainty construals which heighten feelings of threat.

The experience of agency may also be related to modal notional categories important to the expression of future time reference. Patients suffering from anxiety and depression often report their sense of agency is constricted by self doubt, feelings of helplessness, or resignation to the inefficacy of action. This may interact with English grammar to produce higher proportions of low-certainty FTR constructions in sufferers of anxiety and depression. There are lots of ways people can talk about the future, and different reasons for doing so. A widely-used framework is to break down these reasons into schedule-based, prediction-based, and intention-based FTR (Dahl, 1985, Dahl, 2000). In schedule-based FTR people talk about well-known scheduled events, e.g. *The train arrives at 6*; in intention-based FTR people talk about their own intentions and plans for future action, e.g. *I’m coming over later*; and in prediction-based FTR people make predictions about less knowable events, e.g. *The Cubs can probably win tomorrow*.^7^ These categories index modal notions of certainty and uncertainty. For instance, most intention-based FTR is higher certainty than prediction-based FTR. English FTR grammar is sensitive to these distinctions. Critically, it is obligatory in English to use either a future tense construction or a modal verb for prediction-based FTR, e.g. *That coin could/may/might/should/would/shall/will/is going to land on heads*. With the exception of the future tense constructions, these generally express low-certainty modality. In contrast to this, modal/tense marking may be eschewed in schedule-based, and some intention-based FTR statements. For instance, *The show is at 5* (schedule), or *I’m leaving her after Easter* (intention) are acceptable, while *?The coin is landing/?lands on heads tomorrow* (prediction) is not.^8^

The point here is that the tendency to form linguistic utterances about one’s own intentions may be bound up with feelings of agency. If sufferers of anxiety and depression feel a lack of agency, this may leave them more dependent on external loci of control. They may therefore feel at the mercy of unpredictable, capricious, and uncertain forces, and be more likely to form prediction-based FTR statements. This could interact with English grammar and result in higher use of low-certainty language. Conversely, if sufferers of anxiety and depression experience reduced agency, this may imply low-certainty representations of future events and lead directly to increased use of low-certainty modal terms.

### 4.2 Construal level theory: Psychological distance, abstraction, and FTR

A second theoretical perspective might also be brought to bear on the results. Construal Level Theory (CLT) posits that “distant” events are represented in similar ways across different modalities, including space, time, probability, and social familiarity. The general term for cross-modal distance is “psychological distance”. CLT claims there is a relationship between psychological distance, and concreteness and abstraction. In this context, “concreteness” refers to things that can be physically perceived (touched, seen, etc.), and “abstraction” refers to things which cannot. CLT claims that psychologically distal events are represented more abstractly, and psychologically proximal events are represented more concretely. For instance, socially distal (an alien compared with a brother), spatially distal (Mars compared with London), probabilistically distal (winning the lottery compared with eating breakfast tomorrow), and temporally distal (the Stone Age compared with yesterday) events are represented less in terms of concrete sense modalities and more in terms of abstract schemas (Trope and Liberman, 2010). Reciprocally, it is hypothesised that abstraction cues expectations of psychological distance, and concreteness expectations of psychological proximity (Trope and Liberman, 2010; for critical perspectives, see Trautmann, 2019). The value of CLT is that its claims provide an empirically supported link between psychological distance and areas of cognition as diverse as moral orientation (Eyal and Liberman, 2012), self-perception (Liberman and Förster, 2009), emotionality (Woltin et al., 2011), tolerance (Luguri et al., 2012), conformity (Ledgerwood and Callahan, 2012), creativity (Ledgerwood and Callahan, 2012), and aesthetic response (Durkin et al., 2020).

Crucially, many outcomes in the present study involved representations hypothesised by CLT to be represented in terms of psychological distance. Specifically, modal notions of high and low certainty may map onto probability. For instance, strong modals such as *must* and *will* indicate high-probability outcomes (p→1), while weak modals such as *could, may* and *might* indicate indeterminate probability outcomes (p≈0.5) (on connections between modality and scalar notions of probability, see Lassiter, 2015, Moss, 2015, Santorio, Romoli, 2017). Additionally, future and past time reference are generally temporally distant compared with present time reference, and time horizons are a concrete measure of temporal distance.

There is evidence that depression, especially, is associated with overly-abstract representations (Gjelsvik, Lovric, Williams, 2018, Ratcliffe, 2015), and can be ameliorated by exposure to concrete sensory stimuli (Takano, Tanno, 2010, Watkins, Taylor, Byng, Baeyens, Read, Pearson, Watson, 2012, Werner-Seidler, Moulds, 2012). Comparable claims have also been made for anxiety (Goldwin, Behar, 2012, White, Wild, 2016). If so, individuals with anxiety and depression may experience the world in a manner that makes it difficult to resolve mental representations into concrete forms, or may give disproportionate attention to abstract domains. If there is a relationship between abstraction and psychological distance, this might explain our results.

Redditors in mental health condition were more likely to make non-present time reference compared with controls. If sufferers of anxiety and depression represent experience more abstractly, CLT predicts this will result in more frequent non-present time reference, because non-present time reference is predicted to involve more abstraction. In fact, this claim is supported by the finding that references to the past and future involved more abstract language than references to the present in a sample of blog posts (Thorstad and Wolff, 2019b).

Additionally, redditors in the mental health condition exhibited shortened time horizons relative to controls. This seems out of keeping with the CLT prediction that high abstraction should result in reference to more distal time frames. However, a crucial point is that we found higher use of low-certainty language in the mental health condition. Both anxiety and depression are associated with the experience of uncertainty, i.e. all possible outcomes may be represented as equally likely and are thus unpredictable (Carleton, Mulvogue, Thibodeau, McCabe, Antony, Asmundson, 2012, Davis, Neta, Kim, Moran, Whalen, 2016, Grillon, Baas, Lissek, Smith, Milstein, 2004, Grupe, Nitschke, 2013, Hirsh, Mar, Peterson, 2012, Ouimet, Gawronski, Dozois, 2009, Saulnier, Allan, Raines, Schmidt, 2019). In our data, sufferers of anxiety and/or depression exhibited decreased time horizons, but increased low-certainty language. A possible explanation is that the future events which preoccupy sufferers of anxiety and depression may be not so proximal as to be easily predictable or so distal as to be irrelevant. In other words, they may be in the “Goldilocks” zone where worries are relevant.

A further point is CLT maintains that low probability events are associated with more abstract representations. The idea is that people use more abstract language to talk about low-certainty events, and *vice-versa.* For instance, compare *I am going to replace a piston* with *I am going to replace a component*. The latter is more abstract and evinces less certainty. Similarly, compare *I live in a small brown stone walk-up in Queens with a green front door and my tabby cat, Jefferson* with *If I win the lottery, I’ll buy a big mansion*. The latter is lower probability and more abstract. If individuals with anxiety and depression construe experience more abstractly (Gjelsvik, Lovric, Williams, 2018, Goldwin, Behar, 2012, Ratcliffe, 2015, White, Wild, 2016), this might itself drive low-certainty FTR language.

### 4.3 Therapeutic applications

Therapeutic interventions might be developed based on the findings. Correlations between language use and mental unwellness provide deepened evidence of the power of discursive constructionism. Discursive constructionism is a theoretical framework which has become widely-used by clinical practitioners over the last several decades. In this view, systems exist not in external reality, but within “language and communicative action” (Anderson and Goolishian, 1988, p. 373) and so these structures “are locally determined through dialogical exchange” (Anderson and Goolishian, 1988, p. 373). As John Austin puts it, words “do things” (1962). That is, the manner in which we talk about things can change how they are construed and experienced. In this perspective, people in anxious and depressed states discursively reshape the objects of their concern. The power of discursive constructionism is that by tuning into the subtleties of language use, therapists can exert influence in an intentional direction. From this perspective, the present results support discursive constructionist approaches by suggesting that illness experiences are embedded in language use patterns. This begs the question: Decoupled from their linguistic correlates, would the phenomenology of anxiety and depression still be the same? Therapeutic intervention could encourage people to use language which expresses higher agency, more certainty, or expanded temporal horizons. These simple interventions could help treat anxiety and depression.

### 4.4 Limitations

A limitation of the present study is that posting comments to online forums dedicated to the discussion of anxiety and depression is a unreliable diagnostic criterion. Any conclusions about the linguistic habits which may characterise these mood disorders should be made cautiously. Additionally, the control condition in Study 1 was designed to collect a sample of representative contemporary social-media English usage. It is possible that the differences we identified would result from comparing *r/All* posters to posters to *any* subreddits dedicated to the discussion of personal growth and change (likely to be central to mental-health-focussed subreddits). Future research might investigate this by constructing a control sample from subreddits dedicated to exercise, musical practice, or education. It seems plausible that discussion of personal growth would involve increased time reference and use of bouletic modal verbs (e.g. *want*). However, it is difficult to explain every finding under such an account. If anything, discussion of personal growth might plausibly result in increased time horizons and decreased low-certainty language, rather than increased uncertain language, decreased time horizons, which is what we found in the mental health condition. Additionally, Study 2 established depression and anxiety using a demonstrably-reliable psychometric tool. The isomorphism between increased temporal distance in anxious participants (Study 2) and decreased time horizons in the mental health condition (Study 1) suggests the Study 1 results picked up on stable features of how anxious individuals relate to future time.

### 4.5 Conclusions

Anxiety and depression continue to affect the lives of many (Dewa and McDaid, 2011). By implementing descriptive big-data analyses, we used the information available on social media to develop novel insights into the habits of speech and thought which may characterise anxiety and depression. We developed a novel tool which can help researchers characterise patterns in linguistic time reference—the FTR classifier. Using this in combination with other natural language processing tools, we showed that redditors who had once posted to forums dedicated to the discussion of anxiety and depression made more frequent past and future time reference, and used a different distribution of FTR type. They used more bouletic, more low-certainty, more deontic, and fewer future tense constructions. While these effects were small, they might be further developed as markers of these disorders. Additionally, we found posters to mental health forums exhibited contracted time horizons relative to control. This motivated a mediation analysis in Study 2 which found that subjective representations of future temporal distance mediated a relationship between anxiety and temporal discounting. Anxious participants construed future events as more distant and were therefore less likely to wait for future rewards. We then outlined why these results might be expected from two theoretical perspectives: functionalist approaches to clinical psychology and construal level theory.

If progress is to be made in understanding and treating these disorders, new markers need to be explored which can help develop more accurate diagnostic criteria, and new tools need to be developed to do this. Predictive approaches to online social data should be complemented by descriptive ones which allow insights from the analysis of social-media data to be tested in controlled environments and applied in novel therapies. Our results suggest that such approaches may yield fruitful insights.

## Data and Code Availability Statement

The authors are committed to open science and have made data and code available at appropriate open-access repositories. The datasets generated and analysed in this article are available in the Open Science Framework repository at https://osf.io/zwmv5/ (Study 1); and https://osf.io/56rtm/ (Study 2). Code for the FTR and FTR-type classifiers are available on Github at https://github.com/cbjrobertson/ftr_classifier/tree/natural_ftr (specifically at commit ceb0d221c32fa41f29ea112504e9a2bed7163ccd). Analysis scripts are available on request to the first author.

## CRediT authorship contribution statement

**Cole Robertson:** Conceptualization, Methodology, Software, Data curation, Writing – original draft, Writing – review & editing, Formal analysis, Investigation, Visualization, Project administration, Funding acquisition. **James Carney:** Methodology, Writing – original draft, Project administration, Funding acquisition. **Shane Trudell:** Writing – original draft.

## Declaration of Competing Interest

The authors declare that they have no known competing financial interests or personal relationships that could have appeared to influence the work reported in this paper.

## Data Availability

We have shared a link to our data (https://osf.io/nqh9r/) in the main text of the manuscript (Section 2.1.4).

## Appendix A Supplementary materials

Supplmentary materials S1 Supplementary Raw Research Data. This is open data under the CC BY license http://creativecommons.org/licenses/by/4.0/ Supplmentary materials S1

## Footnotes

1 Other: for so-called gnomic truth statements, e.g. *It rains a lot in Oxford.*

2 For indeterminable cases.

3 The default values. This means the model was trained on 80% of the data, and its performance was then evaluated against 20% withheld data. Ten iterations means data were passed over ten times during training. Dropout rate refers to the practice of letting a set proportion of network nodes drop out during training iterations, which can prevent over-fitting by limiting the influence of single nodes. Exclusive categories means sentences cannot be both past and future time reference.

4 Design effect scores Kish (1965) were calculated for other potential sources of group-wise standard error inflation, i.e. multiple posts from a single subreddit or multiple sentences from a single post. This analysis indicated these grouping factors could be ignored, see SM.

5 We note that setting $\alpha_{crit}$ to.05 is arbitrary and join researchers who take the view that there is not much substantive difference between p-values which closely approach $\alpha_{crit}=0.05$ from either side (e.g. Thiese et al., 2016).

6 In these parameter summaries, “pp” reports the probability that the posterior probability distribution of the parameter matches the direction of the hypothesis. In contrast to frequentist p values, pp>0.95 indicates evidence for the parameter matching the prediction exceeds 95% (Bürkner, 2017). Similarly, CI is the Bayesian Credibility Interval, see Fig. 7.

7 On the basis of the Study 2 results, we might add desire-based FTR to this schema, given its high proportion of total FTR statements.

8 Unacceptable language usage is marked with a preceding question mark.
