# Supplementary material for: Language about the future on social media as a novel marker of anxiety and depression: A big-data and experimental analysis
Source: Curr Res Behav Sci. 2023;4:None. doi: 10.1016/j.crbeha.2023.100104 (PMC10308542; doi:10.1016/j.crbeha.2023.100104)
Supplement: Supplmentary materials S1 — Supplementary Raw Research Data. This is open data under the CC BY license http://creativecommons.org/licenses/by/4.0/ [file mmc1.pdf]

# Supplementary Materials for

Language about the future on social media as a novel marker of anxiety and depression:

A big-data and experimental analysis

Cole Robertson*

Emory University

James Carney

The London Interdisciplinary School

Shane Trudell

Nightingale Counselling

Author Note

*corresponding author: 201 Dowman Dr, Atlanta, GA 30322,
cole.robertson@emory.edu

# 1 Installation instructions for the FTR classifier

The version of the FTR classifier which was used in this publication can be installed from the `natural_ftr` branch of the FTR classifier Github repository at `https://github.com/cbjrobertson/ftr_classifier/tree/natural_ftr`. See the `README.md` for instructions on installation and use.

# 2 Description of Twitter data on which the time-reference classifier was trained

A sample of $n = 3000$ future time reference (FTR) annotations for the time-reference classifier were initially drawn from a dataset of tweets. We then added $n = 1002$ examples from the Reddit data which forms the basis of Study 1. We describe the Twitter data here. Data were downloaded from Twitter using the open-source Python package *twint* (Poldi et al., 2019) in June 2019, as part of research involving the prediction of football match outcomes from Twitter data (in prep.). Our starting point was the publicly-available "Football matches odds" dataset (Silvas, 2018), which can be downloaded from Kaggle. The dataset contains information on football match outcomes between December 2016 and May 2018 for approximately 32,000 matches, 500 leagues, and 4,500 teams. Data includes home and away team names and the date of each match. Boolean operators are permitted in *twint*, so we created search terms by concatenating home and away team names with the Boolean operator AND, i.e. [$\text{NAME}_{home}$] AND [$\text{NAME}_{away}$]. Dates were constrained to the two-week period immediately preceding each match date, and we downloaded only English tweets. This allowed us to download FTR tweets made in advance of each match. This resulted in a dataset of $N = 141,428$ tweets made about upcoming football matches. The earliest tweet in this data was made on 10 December 2016 and the latest was made on 22 May 2016. These data were used as the source for the initial $n = 3000$ FTR annotations on which the time-reference classifier was trained.

**2.0.1 Statistical approach.** To test our hypotheses in Study 1, we needed to ensure that standard errors were not inflated by the complexity of the data structures

involved. The data contained multiple sentences from individual posts, multiple posts from individual redditors, and multiple posts and redditors across various subreddits. Using ordinary regressions which assume observations are independent might therefore have risked underestimating actual standard errors and increasing Type I error rates. Our statistical solution to this issue was to calculate "design effect" (*deff*) for each potential source of within-group variation (redditors, posts, subreddits) for each dependent variable in the study. We used *deff* to ascertain whether it was necessary to employ mixed regression techniques to address complex data structures. Kish (1965) defines the *deff* of some sample statistic as "the ratio of the actual variance of a sample of the variance of a simple random sample of the same number of elements" (p. 258). In mixed regression modelling, *deff* can be calculated using the Intraclass Correlation Coefficient (ICC) and the average group size, $c$, such that (Muthén & Satorra, 1995):

$$deff = 1 + (c - 1) \times ICC \tag{1}$$

where $ICC$ is the ratio of within-group to total variance. *Deff* is sensitive to either high $ICCs$ or high group sizes. We calculated *deff* for each grouping factor and each dependent variable, using the entire Study 1 data. For all dependent variables, *deff* scores for posts were low, $1.01 \leq deff_{posts} \leq 1.12$, as were *deff* scores for subreddits, $1.03 \leq deff_{subred} \leq 1.27$. However, *deff* scores for redditors tended to be higher, $1.1 \leq deff_{red} \leq 4.48$. We were therefore justified in ignoring group-wise correlation within posts and subreddits, but estimated multilevel regressions with random intercepts clustered by redditor. This approach allowed us to avoid over-inflating standard errors to within acceptable criteria (i.e. $\approx< 10\%$ according to *Table 3* in Lai & Kwok, 2015).

## 3 FTR-type proportions without dominated future tense

The FTR-type classifier does not, by default, count expressions which use the future tense in combination with modal modifiers (e.g. *possibly*) or mental state predicates (e.g. *think*) as future tense. For example, *I think it will rain tomorrow* and *It*

*will possibly rain tomorrow* would be classified as other-low-certainty and not also future tense, even though both use *will.* We comment in the main text that FTR-type usage proportions indicate English FTR is characterised by a mixture of FTR types, rather than a preponderance of future tense constructions. It was therefore appropriate to check whether this was an artefact of the modal dominance scheme. As such, we calculated usage proportions using non-dominated future tense, which counts any use of *will, shall,* or *be going to* as future tense, regardless of what other keywords are additionally present, *Fig. 1.* Future tense use was higher than the dominated proportions reported in the main text, *Fig.* **??**, but the overall pattern was similar. This suggests English FTR is characterised by a mixture of different FTR types, as we conclude in the main text.

## 4 DASS-21 items

Items for the full DASS-21 are given in *Table 1.* These are from Lovibond and Lovibond (1995). Participants rated these using a Likert scale between 'did not apply to me at all" (0), "applied to me to some degree, or some of the time" (1), "applied to me to a considerable degree, or a good part of time" (2), and "applied to me very much or most of the time" (3). Depression and anxiety scores were 2x the sum of scores within each sub-scale.

## 5 Controlling for choice stochasticity

To test whether a difference in random choice strategy in the intertemporal choice task among anxious and/or depressed participants drove results, we first established choice randomness by calculating the proportion of choices (per participant) which did not agree with the predicted choices under the estimated $k$—"error proportion" (Franco-Watkins, Pashler, & Rickard, 2006). We then added this as a second mediator

in the mediation model:

$$\begin{aligned} subj.dist_i &= \lambda_1 + \alpha_1 anx_i + \alpha_2 dep_i + e_{1i} \\ err.prop_i &= \lambda_2 + \alpha_3 anx_i + \alpha_4 dep_i + e_{2i} \\ log_e(k)_i &= \lambda_3 + \tau_1' anx_i + \tau_2' dep_i + \beta_1 subj.dist_i + \beta_2 err.prop_i + e_{3i} \end{aligned} \tag{2}$$

where $\lambda_{1-3}$ are intercepts, $\alpha_{1-4}$ are slope coefficients for the effects of anxiety and depression on mean subjective temporal distance and error proportion, $\tau'_{1,2}$ and $\beta_{1,2}$ are slope coefficients for the effects of anxiety, depression, mean subjective distance, and error proportion on temporal discounting ($log_e(k)$), and $e_{1-3}$ are error terms. See *Fig. 2* for a conceptual diagram.

This allowed us to test the hypotheses that anxious and/or depressed participants used a different random choice-answering strategy, which in turn impacted discounting ($log_e(k)$). It did not. Depression and anxiety scores did not predict error proportion *Table 2*. This meant that the two-tailed 95% credibility interval for both indirect effects via error proportion contained 0 (anxiety: $Est. = -0.02,\ CI_{95\%} = [-0.04, 0]$; depression: $Est. = 0,\ CI_{95\%} = [0, 0.01]$). The same was true of the total effects (anxiety: $Est. = 0.04,\ CI_{95\%} = [-0.02, 0.11]$; depression: $Est. = -0.01$, $CI_{95\%} = [-0.06, 0.03]$). This indicates that depression and anxiety did not impact discounting via choice stochasticity.

The hypothesis tests conducted in the main text remained qualitatively unchanged for the most part: The only difference was the direct effect of anxiety was now significant, $Est. = 0.06,\ CI_{90\%} = [0.01, 0.12],\ pp = .962$. This indicates that, when controlling for choice stochasticity, anxiety may have caused increased discounting independent from the indirect effect via subjective temporal distance. The indirect and total effects of anxiety via subjective temporal distance remained unchanged (indirect: $Est. = 0.04,\ CI_{90\%} = [0.02, 0.06],\ pp > .999$; total: $Est. = 0.1,\ CI_{90\%} = [0.05, 0.15]$, $pp = .999$). As in the main text, no effects of depression were significant (indirect: $Est. = 0,\ CI_{90\%} = [-0.01, 0],\ pp = .19$; direct: $Est. = -0.02,\ CI_{90\%} = [-0.05, 0.02]$, $pp = .22$; total: $Est. = -0.02,\ CI_{90\%} = [-0.06, 0.02],\ pp = .17$). Together, these results

indicate that, on the whole, results were not affected by controlling for choice stochasticity.

## Tables

**Table 1**

*DASS-21 items*

| sub-scale | question |
|---|---|
| depression | I couldn't seem to experience any positive feeling at all |
| | I found it difficult to work up the initiative to do things |
| | I felt that I had nothing to look forward to |
| | I felt down-hearted and blue |
| | I was unable to become enthusiastic about anything |
| | I felt I wasn't worth much as a person |
| | I felt that life was meaningless |
| anxiety | I was aware of dryness of my mouth |
| | I experienced breathing difficulty (e.g. excessively rapid breathing, breathlessness in the absence of physical exertion) |
| | I experienced trembling (e.g. in the hands) |
| | I was worried about situations in which I might panic and make a fool of myself |
| | I felt I was close to panic |
| | I was aware of the action of my heart in the absence of physical exertion (e.g. sense of heart rate increase, heart missing a beat) |
| | I felt scared without any good reason |
| stress | I found it hard to wind down |
| | I tended to over-react to situations |
| | I felt that I was using a lot of nervous energy |
| | I found myself getting agitated |
| | I found it difficult to relax |
| | I was intolerant of anything that kept me from getting on with what I was doing |
| | I felt that I was rather touchy |

**Table 2**

*Model parameters for model with error proportion included*

| outcome | predictor | parameter name | Estimate | SE | lower-95% CI | upper-95% CI | Rhat |
|---|---|---|---|---|---|---|---|
| subjective temporal distance | intercept | $\lambda_1$ | 28.23 | 1.08 | 26.15 | 30.36 | 1.00 |
| error proportion | intercept | $\lambda_2$ | 0.06 | 0.00 | 0.05 | 0.06 | 1.00 |
| logk | intercept | $\lambda_3$ | -4.05 | 0.44 | -4.91 | -3.18 | 1.00 |
| subjective temporal distance | anxiety | $\alpha_1$ | 0.98 | 0.18 | 0.63 | 1.33 | 1.00 |
| subjective temporal distance | depression | $\alpha_2$ | -0.12 | 0.13 | -0.37 | 0.13 | 1.00 |
| error proportion | anxiety | $\alpha_3$ | 0.00 | 0.00 | 0.00 | 0.00 | 1.00 |
| error proportion | depression | $\alpha_4$ | 0.00 | 0.00 | 0.00 | 0.00 | 1.00 |
| logk | subjective temporal distance | $\beta_1$ | 0.04 | 0.01 | 0.02 | 0.06 | 1.00 |
| logk | anxiety | $\tau_1$ | 0.06 | 0.03 | -0.01 | 0.13 | 1.00 |
| logk | depression | $\tau_2$ | -0.02 | 0.02 | -0.06 | 0.02 | 1.00 |
| logk | error proportion | $\beta_2$ | -6.63 | 3.81 | -13.97 | 0.90 | 1.00 |

## Figures

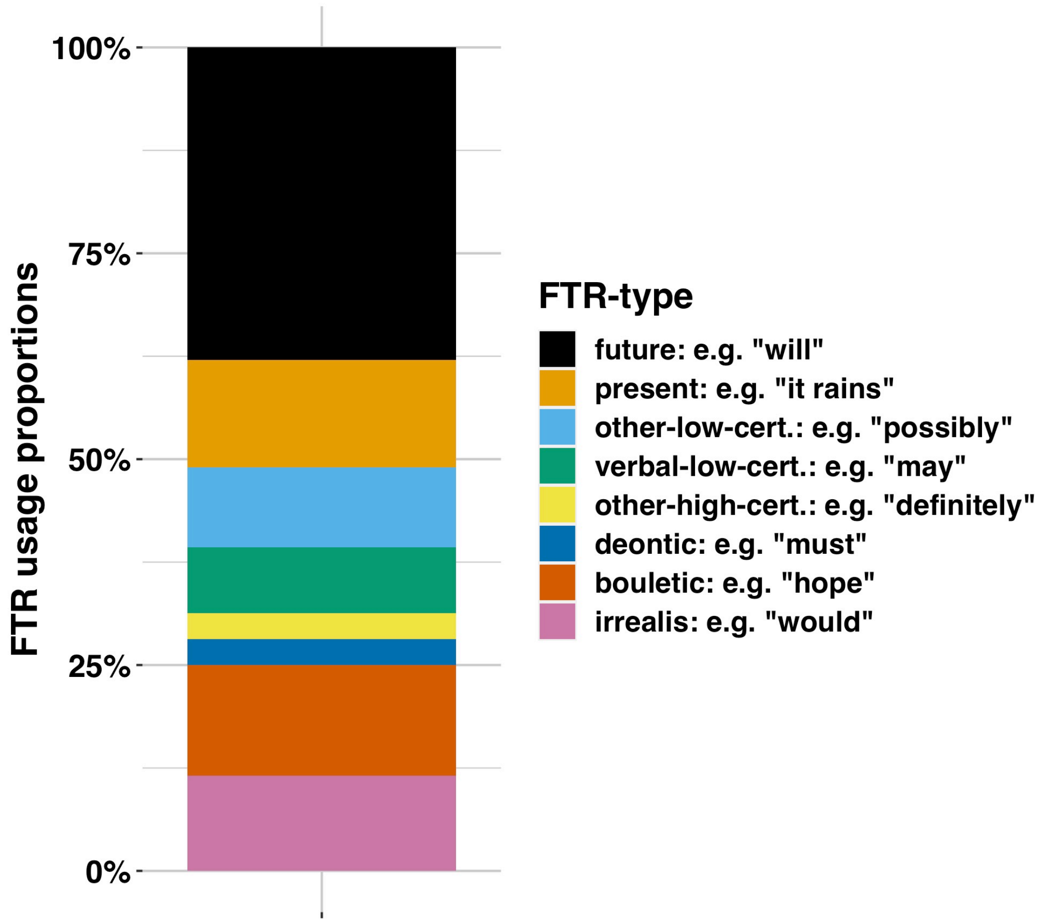


*Figure 1.* Differences in probability of using different FTR types when referring to the future as a function of condition in Study 1. Data are the same as in *Fig.* **??** in the main text.

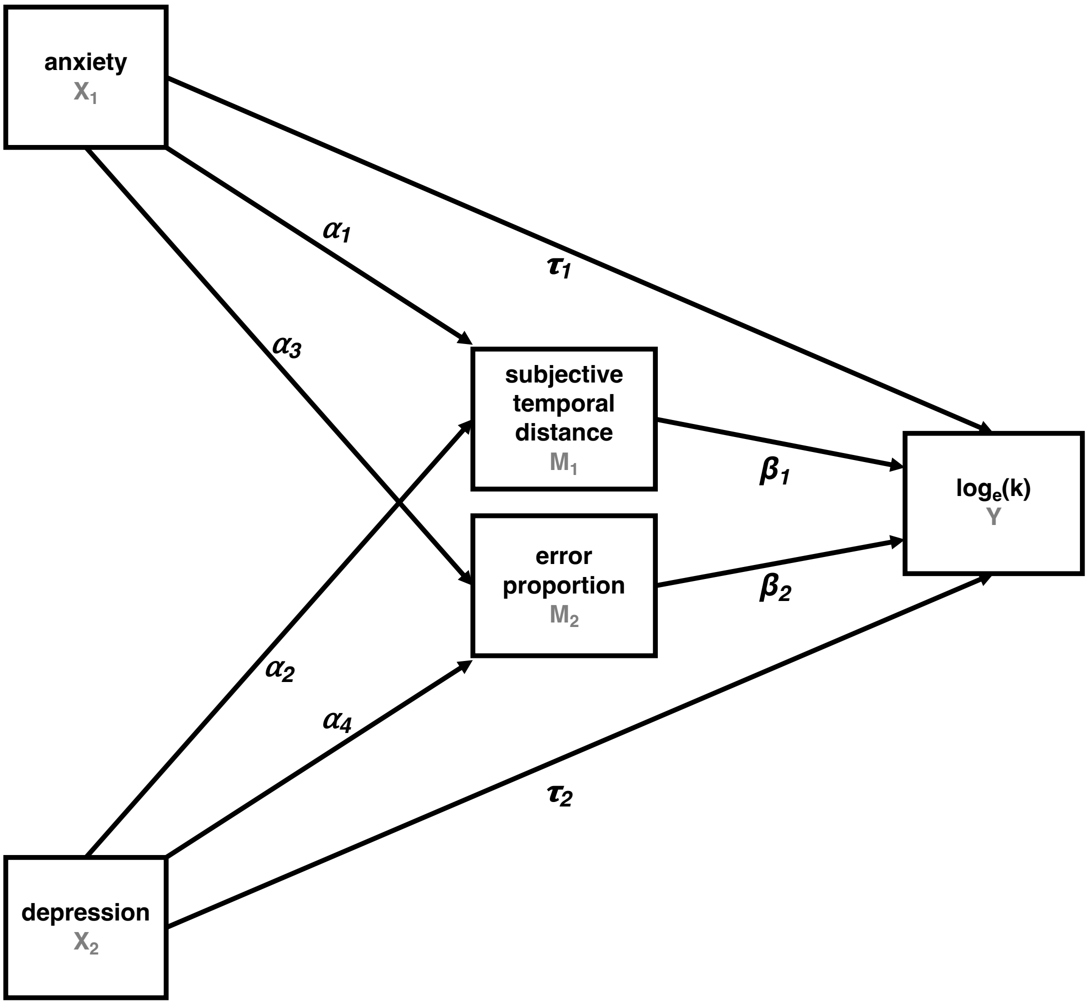


*Figure 2.* Conceptual model diagram of model with error proportion included. Parameter names correspond to *Table 2*.

## 6 References

Franco-Watkins, A. M., Pashler, H., & Rickard, T. C. (2006). Does working memory load lead to greater impulsivity? Commentary on Hinson, Jameson, and Whitney (2003). *Journal of Experimental Psychology: Learning Memory and Cognition*, *32*(2), 443–447. doi: 10.1037/0278-7393.32.2.443

Kish, L. (1965). *Survey sampling.* New York: John Wiley & Sons Ltd.

Lai, M. H., & Kwok, O. M. (2015). Examining the rule of thumb of not using multilevel modeling: The "design effect smaller than two" rule. *Journal of Experimental Education*, *83*(3), 423–438. doi: 10.1080/00220973.2014.907229

Lovibond, S. H., & Lovibond, P. F. (1995). *Manual for the Depression Anxiety and Stress Scales* (2nd ed.). Sydney: Psychology Foundation.

Muthén, B. O., & Satorra, A. (1995). Complex sample data in structural equation modeling. *Sociological Methodology*, *25*, 267–316. doi: 10.2307/271070

Poldi, F., Zacharias, C., Levin, M., Hpiedcoq, Urbanski, A., Nestor75, ... Aldou (2019). *Twint.* Retrieved from `https://github.com/twintproject/twint`

Silvas, E. (2018). *Football matches odds: Changes in football winning odds - 32k+ matches, 500+leagues, 4.5k+ teams.* Retrieved from `https://www.kaggle.com/eladsil/football-games-odds`
